# Sustainable ZnO/Zn_3_(PO_4_)_2_ Nanoparticles Synthesized from Coconut-Derived Media Incorporated into Bioactive ALG/PVA Hydrogel Dressings

**DOI:** 10.3390/gels12030243

**Published:** 2026-03-13

**Authors:** Alexandra Cătălina Bîrcă, Alexandra Cristina Burdușel, Adelina-Gabriela Niculescu, Carmen Curuțiu, Alina Maria Holban, Alexandru Mihai Grumezescu, Ariana Hudiță, Bianca Gălățeanu, Bogdan Severus Gaspar, Alfred Najm

**Affiliations:** 1 Department of Science and Engineering of Oxide Materials and Nanomaterials, National University of Science and Technology Politehnica Bucharest, 011061 Bucharest, Romania; alexandra.birca@upb.ro (A.C.B.); alexandra.burdusel@upb.ro (A.C.B.); adelina.niculescu@upb.ro (A.-G.N.); 2 Research Institute of the University of Bucharest—ICUB, University of Bucharest, 050657 Bucharest, Romania; carmen.curutiu@bio.unibuc.ro (C.C.); alina.m.holban@bio.unibuc.ro (A.M.H.); ariana.hudita@bio.unibuc.ro (A.H.); bianca.galateanu@bio.unibuc.ro (B.G.); 3 Faculty of Biology, University of Bucharest, 030018 Bucharest, Romania; 4 Department of Surgery, Carol Davila University of Medicine and Pharmacy, 8 Eroii Sanitari, Sector 5, 050474 Bucharest, Romania; bogdangaspar2005@yahoo.com (B.S.G.); alfred.najm@yahoo.ro (A.N.); 5 Emergency Hospital Floreasca Bucharest, 8 Calea Floreasca, Sector 1, 014461 Bucharest, Romania

**Keywords:** green synthesis, zinc-based nanoparticles, alginate, poly(vinyl alcohol), spirulina, aronia, antimicrobial, biocompatibility

## Abstract

The adaptive nature of bacteria and their increasing resistance to conventional therapies demand alternative strategies to effectively control wound infections. At the wound site, dynamic biological processes are easily disrupted by microbial colonization, compromising normal healing. In this study, Zn-based nanoparticles composed of zinc oxide (ZnO) and zinc phosphate (Zn_3_(PO_4_)_2_) were synthesized via a green route using coconut milk and coconut water as biological media. Although ZnO formation via zinc hydroxide intermediates was initially targeted, structural analyses revealed a multiphase Zn-based system resulting from interactions between Zn^2+^ ions and naturally occurring phosphate species in the coconut-derived sources. The resulting material was incorporated into sodium alginate/poly(vinyl alcohol) hydrogel dressings, further enhanced with spirulina and aronia powders. Physicochemical characterization (XRD, SEM, EDS, FTIR), along with swelling and degradation studies, confirmed structural stability and appropriate hydrogel behavior. Antimicrobial testing against *Staphylococcus aureus* and *Escherichia coli* demonstrated a dominant antibiofilm effect of the Zn-based nanoparticles, while botanical additives exhibited moderate, time-dependent activity. Biological evaluation demonstrated good cytocompatibility toward human keratinocytes and murine macrophages, with botanical additives mitigating mild nanoparticle-induced cellular responses.

## 1. Introduction

Throughout life, almost every individual experiences at least one form of physical or chemical damage that results in a wound, defined as a disruption of skin integrity. Such wounds may exhibit different characteristics depending on the severity of the damage and are generally classified as acute or chronic. Although the fundamental healing mechanism follows the same initial stages, hemostasis and inflammation, acute wounds proceed through the natural progression of healing, entering the proliferative stage and subsequently the remodeling process. In contrast, chronic wounds fail to progress beyond the inflammatory stage [1,2,3,4]. At this point, external intervention becomes crucial to support the physiological repair process and overcome factors that impair healing, most notably infections [5,6,7,8]. To assist and enhance wound healing, wound dressings are widely employed. These dressings are essential medical devices in healthcare, and contemporary technological advancements enable them to be tailored to the specific requirements of different wound types. Modern wound care strategies are guided by several key principles that must be fulfilled to achieve complete recovery without adverse or unintended effects [9,10,11,12]. These principles include maintaining a moist environment, ensuring biocompatibility, managing wound exudate, enabling the controlled release of drugs or engineered therapeutic systems, reducing pain, and providing biodegradability [1,13,14,15]. Such criteria support the natural healing cascade and are predominantly met by advanced dressing types such as hydrogels, alginates, hydrocolloids, foams, and films. These materials can be readily engineered and optimized by adjusting formulation parameters and processing conditions, enabling the development of formulations that address the complex needs of difficult-to-heal wounds. This adaptability is largely attributed to the intrinsic physicochemical and therapeutic characteristics of the materials from which these dressings are fabricated [12,16,17,18,19].

Among the most widely used types of wound dressings are hydrogels, which are three-dimensional polymeric networks characterized by their ability to exhibit a high water uptake capacity without compromising structural integrity. Within this category, sodium alginate (ALG)-based formulations are particularly prominent, primarily due to their natural origin, which enhances biocompatibility. ALG hydrogels represent a natural and highly adaptable platform that can be tailored to meet specific wound care requirements [20,21]. They are biocompatible, biodegradable, and possess a high fluid-absorption capacity. Owing to their hydrophilic nature, alginate systems can undergo gelation upon contact with wound exudate, forming a hydrated gel layer at the wound interface. This gel structure promotes moisture retention, provides pain relief, and maintains a flexible protective covering that helps reduce bacterial contamination while supporting the physiological healing process [17,22,23].

To further enhance their physicochemical and biological performance, alginate hydrogels can be combined with other natural substances such as collagen or chitosan, as well as with synthetic yet biocompatible polymers including poly(vinyl alcohol) (PVA), polylactic acid (PLA), and polyvinylpyrrolidone (PVP) [24,25,26]. Among these combinations, the ALG/PVA system has emerged as one of the most extensively investigated, representing the basis for a new class of PVA–ALG wound dressings [27]. PVA itself is widely recognized as a suitable polymer for wound dressing applications due to its excellent biocompatibility, favorable swelling behavior, and controllable degradation profile [28,29]. When combined, ALG and PVA form a composite matrix that meets key wound care requirements, including biocompatibility, biodegradability, moisture retention, swelling capacity, gel-forming ability, and pain reduction [30,31,32]. However, despite these advantageous properties, ALG and PVA alone do not inherently provide sufficient antimicrobial activity against pathogenic microorganisms that may colonize and infect the wound site.

In this context, the functional optimization of wound dressings extends into nanotechnology, a powerful tool in modern medicine, particularly in antimicrobial applications [33]. A wide range of nanoparticles has demonstrated remarkable efficacy in combating bacterial infections [34,35]. Among them, zinc (Zn)-based nanoparticles have attracted considerable attention due to their biological relevance and antimicrobial performance [36,37,38]. Zinc oxide (ZnO) nanoparticles are among the most extensively studied and utilized Zn-based nanomaterials in biomedical applications. They can be synthesized through various chemical, physical, and green routes, resulting in diverse morphologies and particle sizes depending on the synthesis parameters. Both nano- and microscale ZnO particles have demonstrated valuable antimicrobial activity, making them highly relevant for research in wound care and infection control [39,40,41,42]. Another Zn-based material of increasing interest is zinc phosphate (ZnP). Traditionally employed in other fields due to its anticorrosive and electrocatalytic properties, ZnP has more recently gained attention in biomedical research owing to its biocompatibility and demonstrated antimicrobial potential. Like ZnO, ZnP can be synthesized through multiple approaches, each enabling the tuning of structural, morphological, and functional characteristics according to the intended application [43,44,45,46].

In the present study, hydrogel-based wound dressing formulations were developed using an ALG and PVA matrix, and further functionalized with Zn-based nanoparticles comprising a mixed ZnO/Zn_3_(PO_4_)_2_ system. The inorganic phase was synthesized via a green approach using coconut milk and coconut water as biological reaction media to obtain ZnO nanoparticles via an eco-friendly pathway. The aqueous zinc nitrate precursor provides Zn^2+^ ions, which, in the presence of hydroxyl-generating constituents naturally occurring in coconut milk and water, are expected to initially form zinc hydroxide (Zn(OH)_2_) intermediates. Upon thermal calcination, Zn(OH)_2_ undergoes dehydration, yielding crystalline ZnO. However, coconut milk and coconut water also contain trace mineral species, including phosphorus in the form of phosphate-containing compounds. The interaction between Zn^2+^ ions and phosphate species during synthesis can promote the nucleation and growth of ZnP even at relatively low phosphate concentrations. As a result, instead of a single-phase ZnO system, a multiphase Zn-based material composed of ZnO and Zn_3_(PO_4_)_2_ crystallization form of ZnP was obtained. To further enhance the biofunctional performance of the developed dressings, spirulina and aronia powders were incorporated into the polymeric matrix. Spirulina contains phycocyanin and bioactive peptides, whereas aronia is rich in anthocyanins and phenolic acids [47,48,49,50,51,52]. The integration of a green-synthesized multiphase Zn-based system with plant-derived bioactive compounds was, therefore, designed to create a multifunctional wound dressing that combines inorganic antibacterial mechanisms with phytochemical-assisted biological support.

## 2. Results

### 2.1. Results of the Characterization of Zinc-Based Powders (ZnO_M.Coco, ZnO_M.Coco.E, and ZnO_W.Coco) After Thermal Treatment at 400 °C for 3 h

**Figure 1:**
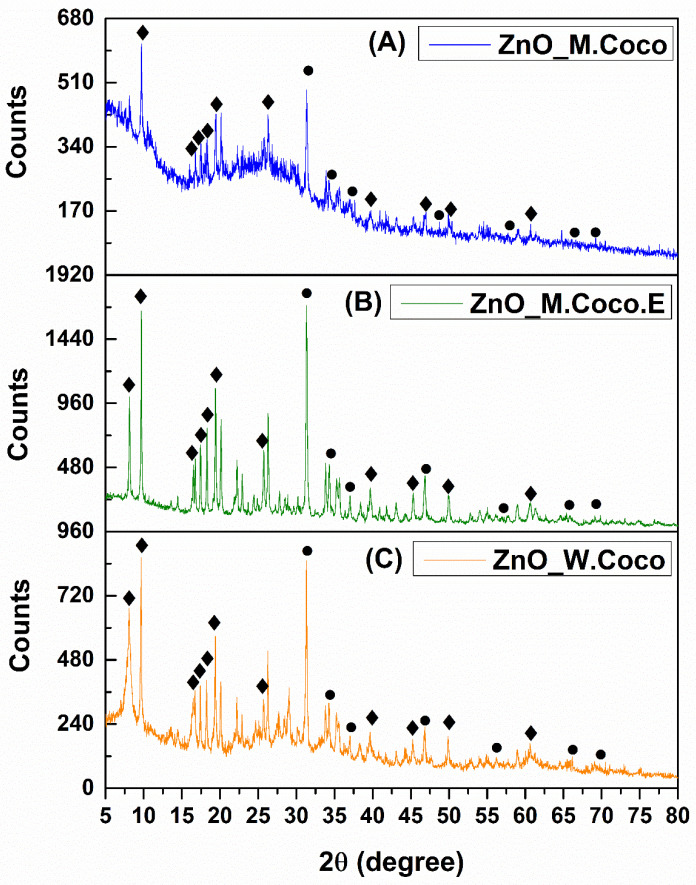
XRD diffraction patterns obtained for ZnO_M.Coco (**A**), ZnO_M.Coco.E (**B**), and ZnO_W.Coco (**C**) after thermal treatment at 400 °C for 3 h. The diffraction peaks corresponding to ZnO are marked with circles (●), while the peaks associated with Zn_3_(PO_4_)_2_·4H_2_O are indicated with diamonds (♦).

Following the synthesis of the three samples obtained via the green method using coconut milk, centrifuged coconut milk (extract), and coconut water, a thermal treatment temperature of 400 °C was selected in order to promote crystallization into zinc oxide and to remove organic compounds originating from the green synthesis sources. This temperature is commonly reported in the literature for the preparation of zinc oxide [53,54,55]. Additionally, an aspect associated with achieving these objectives is the white color expected for the powder after thermal treatment. In the present research activity, after thermal treatment at 400 °C for 3 h, all three powders exhibited a completely black appearance. Figure 1A presents the X-ray diffraction pattern obtained for the ZnO_M.Coco sample after thermal treatment at 400 °C for 3 h.

At first glance, the diffraction pattern indicates the presence of residual organic components, suggesting incomplete removal of the green synthesis-derived organic matter. Nevertheless, the X-ray diffraction pattern reveals multiple diffraction peaks characteristic of two distinct phases: zinc oxide (ZnO) and zinc phosphate (ZnP). The ZnO phase is evidenced by characteristic diffraction reflectionslocated at 2θ values of 31.79°, 34.43°, 36.26°, 47.59°, 56.64°, 66.39°, and 69.10°, assigned to the Miller indices (100), (002), (101), (102), (110), (200), and (201), respectively, and confirmed by the 04-007-1614 reference code from the PDF-ICDD database. The second identified phase corresponds to Zn_3_(PO_4_)_2_·4H_2_O (JCPDS number 33-1474), which is represented by diffraction peaks at 2θ values of 9.71°, 18.30°, 19.41°, 39.68°, 46.95°, 50.00°, and 60.9°, assigned to the Miller indices (020), (011), (040), (171), (371), (402), and (303), respectively [56,57]. Additionally, carbon-related peaks are observed at multiple diffraction angles, suggesting the carbonization of organic compounds derived from the green synthesis sources.

Figure 1B presents the X-ray diffraction pattern obtained for the ZnO_M.Coco.E sample after thermal treatment at 400 °C for 3 h. Although both ZnO_M.Coco and ZnO_M.Coco.E samples were synthesized using coconut milk, a notable difference was observed for the sample prepared using centrifuged coconut milk. Considering that the centrifugation process removed a significant fraction of the fats present in the coconut milk, the thermal treatment at 400 °C for 3 h involved the combustion of fewer organic compounds, resulting in a more crystalline diffraction pattern. In this case as well, the same two phases, namely ZnO and Zn_3_(PO_4_)_2_·4H_2_O, were identified. However, compared to the ZnO_M.Coco sample, the ZnO_M.Coco.E diffraction pattern exhibits two diffraction peaks below 10° 2θ, as well as additional peaks at 16.65°, 17.80°, and 18.22°, corresponding to the Miller indices (200), (210), and (011), respectively, which are associated with the Zn_3_(PO_4_)_2_·4H_2_O phase. The ZnO phase is still identified at the same diffraction angles as in the previous sample. However, these peaks exhibit higher intensities and improved definition, indicating enhanced crystallinity.

The diffraction pattern obtained for the ZnO_W.Coco sample after thermal treatment at 400 °C for 3 h is shown in Figure 1C. When compared with the ZnO_M.Coco and ZnO_M.Coco.E samples, a closer similarity in terms of peak identification can be observed between the ZnO_M.Coco.E and ZnO_W.Coco diffraction patterns. This behavior can be attributed to the lower fat content of the green synthesis source used for the ZnO_W.Coco sample. Nevertheless, the overall appearance and definition of the diffraction peaks indicate that the ZnO_W.Coco sample exhibits a slightly lower degree of crystallinity compared to the ZnO_M.Coco.E sample.

**Figure 2:**
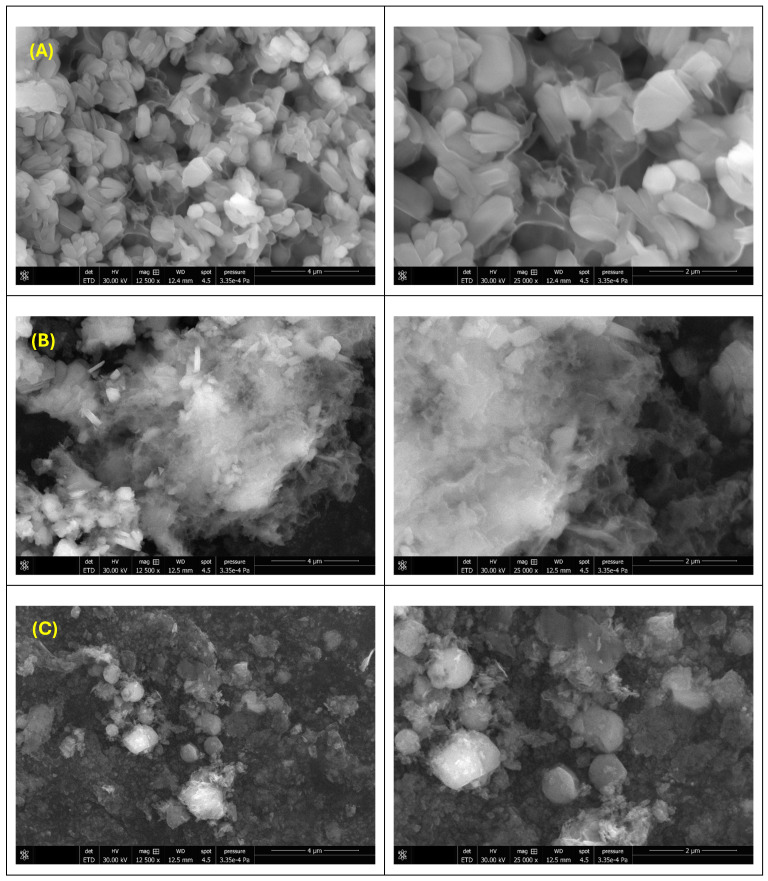
SEM micrographs obtained for ZnO_M.Coco (**A**), ZnO_M.Coco.E (**B**), ZnO_W.Coco (**C**) after thermal treatment at 400 °C for 3 h.

Figure 2 presents the SEM micrographs obtained for the ZnO_M.Coco (A), ZnO_M.Coco.E (B), and ZnO_W.Coco (C) samples after thermal treatment at 400 °C for 3 h. Figure 2A highlights the morphology of the ZnO_M.Coco sample, which is characterized by plate-like particles assembled into small agglomerates, partially covered by a continuous phase associated with residual organic components. The centrifugation of coconut milk prior to synthesis induced noticeable changes in particle morphology, as observed in Figure 2B for the ZnO_M.Coco.E sample. In this case, finer particle morphologies are obtained, forming larger agglomerates in which individual particles cannot be clearly distinguished. The use of coconut water as the green synthesis medium led to a third distinct morphology, identified for the ZnO_W.Coco sample (Figure 2C). This sample exhibits irregular, rounded agglomerates with rough surfaces, decorated with smaller plate-like structures.

**Figure 3:**
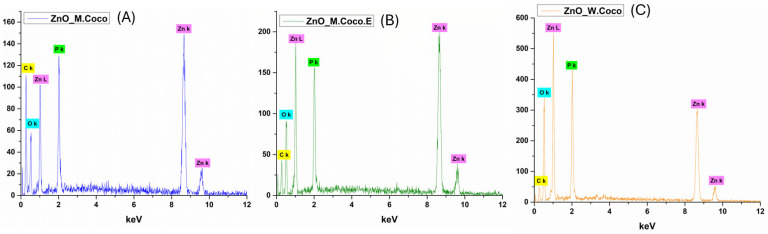
EDS results obtained for ZnO_M.Coco (**A**), ZnO_M.Coco.E (**B**), ZnO_W.Coco (**C**) after thermal treatment at 400 °C for 3 h.

Figure 3 presents the EDS spectra recorded for ZnO_M.Coco (A), ZnO_M.Coco.E (B), and ZnO_W.Coco (C) after thermal treatment at 400 °C for 3 h. For all three samples, the presence of Zn and O is clearly evidenced, confirming the formation of the ZnO phase previously identified by XRD analysis. Additionally, the detection of P in all samples further supports the identification of a secondary ZnP phase accompanying the ZnO phase. Notable differences in elemental composition are observed as a function of the synthesis parameters (see Table 1). In the case of ZnO_M.Coco, a significantly higher C content is detected (51.62 wt%) compared to ZnO_M.Coco.E (28.01 wt%). This difference can be attributed to the centrifugation step applied to coconut milk, which removes a substantial fraction of lipid-rich organic components, thereby facilitating a more efficient combustion of organic residues during thermal treatment. Concomitantly, higher contents of Zn (20.88 wt%) and O (41.02 wt%) are observed for ZnO_M.Coco.E, indicating an enhanced formation of ZnO and ZnP phases. On the other hand, the ZnO_W.Coco sample exhibits the lowest C content and the highest Zn content among all analyzed samples. This behavior is associated with the intrinsically lower lipid and organic content of coconut water compared to coconut milk.

**Figure 4:**
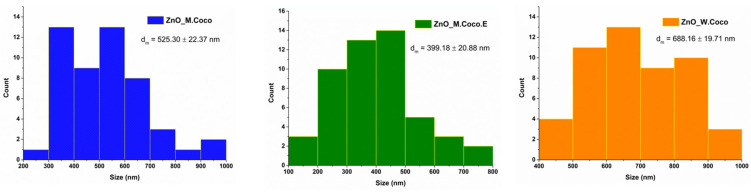
Size distribution histograms obtained for ZnO_M.Coco, ZnO_M.Coco.E, ZnO_W.Coco after thermal treatment at 400 °C for 3 h.

The size distribution histograms are presented in Figure 4. The measurements could be performed more reliably for the ZnO_M.Coco sample due to the morphology of the particles. The particle sizes range between approximately 200 and 1000 nm. For the ZnO_M.Coco.E sample, irregular morphologies are observed, making it difficult to identify and measure individual particles. Therefore, the measurements were performed on the visible aggregated structures, whose sizes ranged from approximately 100 to 800 nm. In the case of the ZnO_W.Coco sample, only the large agglomerates visible in the SEM images were measured; the sizes of these agglomerates range approximately from 400 to 1000 nm.

Considering the results obtained for the three powders after thermal treatment at 400 °C for 3 h, it should be noted that the green synthesis route successfully yielded ZnO, as confirmed by XRD analysis. However, an additional phase accompanying ZnO was also detected, namely tetrahydrate zinc phosphate (Zn_3_(PO_4_)_2_·4H_2_O), which can be attributed to the complex composition of the coconut-derived synthesis media (coconut milk and coconut water), including naturally occurring mineral species. Furthermore, the black appearance of the powders and the diffraction patterns suggested the presence of residual organic/carbonized components originating from the coconut sources. Therefore, the samples were further subjected to a higher-temperature thermal treatment at 800 °C for 2 h.

### 2.2. Results of the Characterization of Zinc-Based Powders (ZnO_M.Coco, ZnO_M.Coco.E, and ZnO_W.Coco) After Thermal Treatment at 800 °C for 2 h

**Figure 5:**
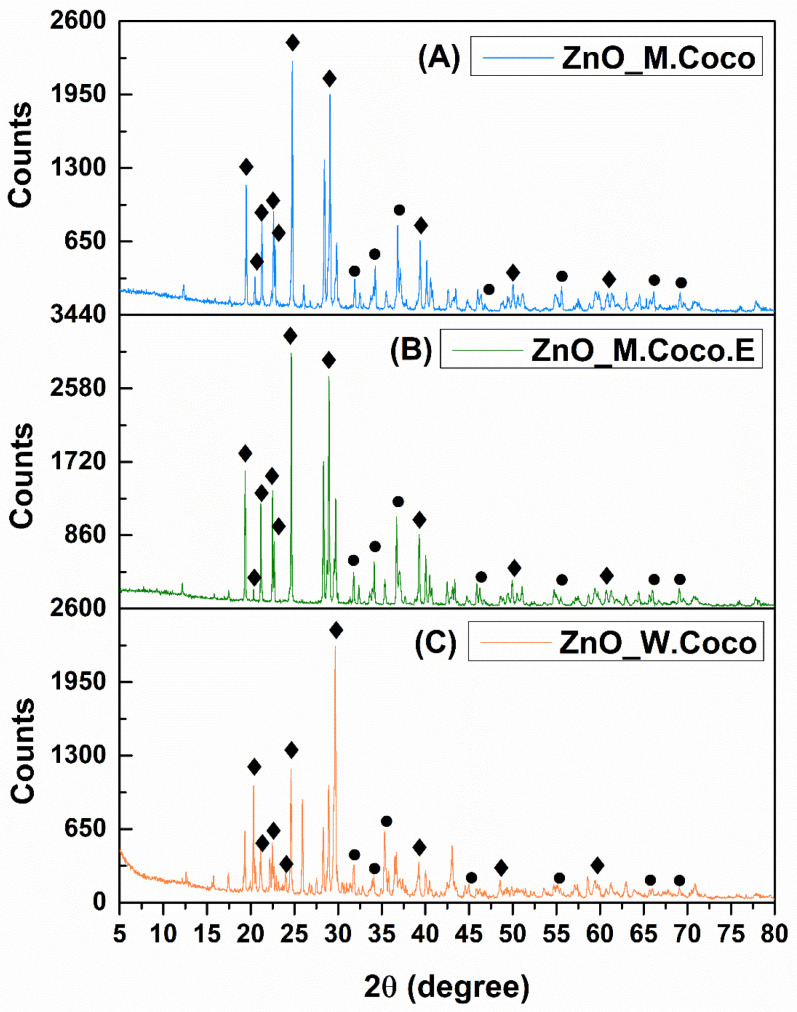
XRD diffraction patterns obtained for ZnO_M.Coco (**A**), ZnO_M.Coco.E (**B**) and ZnO_W.Coco (**C**) after thermal treatment at 800 °C for 2 h. The diffraction peaks corresponding to ZnO are marked with circles (●), while the peaks associated with Zn_3_(PO_4_)_2_ are indicated with diamonds (♦).

The next experimental step involved selecting a significantly higher thermal treatment temperature to promote the complete combustion, rather than mere carbonization, of the organic compounds originating from the green synthesis sources, while still maintaining a temperature that would not negatively affect the ZnO structure and could potentially favor the formation of a single ZnO phase. It should be noted that, following this thermal treatment, all powders exhibited a white appearance. Figure 5A presents the diffractogram of the ZnO_M.Coco powder after thermal treatment at 800 °C for 2 h.

In this case, compared to the diffraction pattern obtained after thermal treatment at 400 °C, it is clear that the sample exhibits a crystalline structure, which correlates with the elimination of carbonized organic compounds and the white coloration of the powders. However, the same two phases, namely ZnO and Zn_3_(PO_4_)_2_, are still identified, although with much better-defined diffraction peaks. The ZnO phase is represented by the same diffraction peaks as previously observed, confirming that the higher temperature did not negatively affect its crystal structure. In the case of ZnP, the previously observed diffraction peak at 9.71° 2θ is no longer detectable. Meanwhile, well-defined peaks appear at 2θ values of 21.51°, 22.17°, 23.30°, and 29.50°, confirmed by the 00-029-1390 reference code from the PDF-ICDD database for the monoclinic crystallization system of Zn_3_(PO_4_)_2_.

The XRD results for the ZnO_M.Coco.E sample are illustrated in Figure 5B. The diffraction pattern shows a strong similarity to that of the ZnO_M.Coco sample, highlighting that this thermal treatment temperature is suitable for the removal of impurities originating from the green synthesis source. In this case as well, compared with the diffraction pattern of the same sample after thermal treatment at 400 °C for 3 h, the two diffraction peaks below 10° 2θ disappear, along with a shift in the peaks below 20° 2θ. As a result, only the peak at approximately 19° 2θ remains in this region, while the other diffraction peaks are located at angles above 20° 2θ corresponding to the anhydrous Zn_3_(PO_4_)_2_ [58]. The ZnO phase is clearly identified by its characteristic diffraction peaks at 31.79°, 34.43°, 36.26°, 47.59°, 56.64°, 66.39°, and 69.10° 2θ, corresponding to the Miller indices (100), (002), (101), (102), (110), (200), and (201), respectively. The diffraction pattern obtained for the sample synthesized using coconut water (ZnO_W.Coco) after thermal treatment at 800 °C for 2 h differs from those of the other two samples synthesized using coconut milk, as evidenced by the presence of a larger number of diffraction peaks as well as by a lower degree of crystallization (Figure 5C).

**Figure 6:**
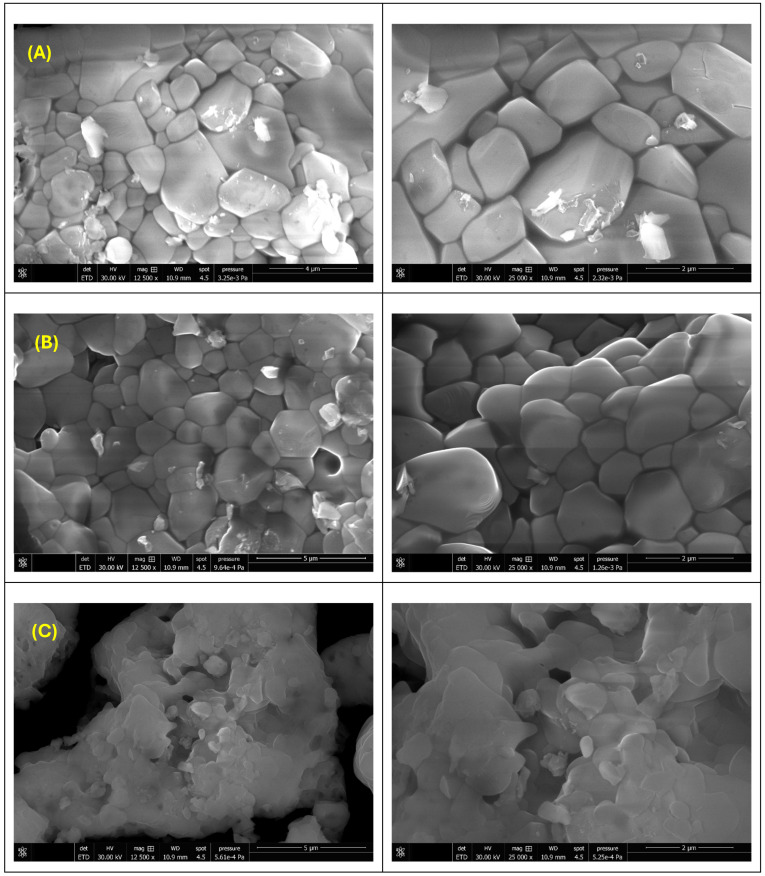
SEM micrographs obtained for ZnO_M.Coco (**A**), ZnO_M.Coco.E (**B**), ZnO_W.Coco (**C**) after thermal treatment at 800 °C for 2 h.

The particle morphology obtained after thermal treatment at 800 °C for 2 h is illustrated in Figure 6A for ZnO_M.Coco, Figure 6B for ZnO_M.Coco.E, and Figure 6C for ZnO_W.Coco. Following this thermal treatment performed at a temperature approximately twice that of the previous treatment, both ZnO_M.Coco and ZnO_M.Coco.E samples exhibit similar morphological features, characterized by irregular particles that have significantly increased in size and coalesced, clearly indicating sintering. The ZnO_M.Coco sample presents large, polygonal grains with well-defined boundaries, while the ZnO_M.Coco.E sample displays slightly more rounded grains. In both cases, the particles form dense, continuous ceramic-like structures with grain sizes in the micrometer range. In the case of the ZnO_W.Coco sample (Figure 6C), a similar sintering process is observed. However, the particle morphology is no longer distinguishable; the material appears as a continuous phase with irregular features.

**Figure 7:**
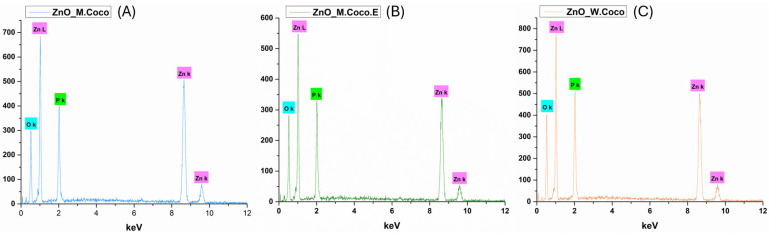
EDS results obtained for ZnO_M.Coco (**A**), ZnO_M.Coco.E (**B**), ZnO_W.Coco (**C**) after thermal treatment at 800 °C for 2 h.

The application of thermal treatment at 800 °C for 2 h induced significant changes in the elemental composition of all three Zn-based powders, as evidenced by the EDS results presented in Figure 7. The EDS spectra corresponding to ZnO_M.Coco (A), ZnO_M.Coco.E (B), and ZnO_W.Coco (C) reveals the exclusive presence of Zn, O, and P, indicating the complete removal of carbonaceous residues originating from the green synthesis routes. These findings are in accordance with the XRD results, confirming the effectiveness of the high-temperature treatment. The quantitative EDS data summarized in Table 2 indicate that ZnO_M.Coco contains relatively high amounts of Zn (42.25 wt%), O (41.28 wt%), and P (16.47 wt%), confirming the simultaneous presence of ZnO and ZnP phases. In the case of ZnO_M.Coco.E, where a more diluted mineral composition was obtained due to the prior removal of fats and associated organic compounds, an increase in O and P content, accompanied by a slight decrease in Zn content, is observed. This trend suggests a more pronounced contribution of ZnP alongside the ZnO phase. Similarly, ZnO_W.Coco exhibits the highest P content (21.25 wt%) together with elevated O levels (53.25 wt%), further supporting the formation of ZnP species in addition to ZnO.

**Figure 8:**
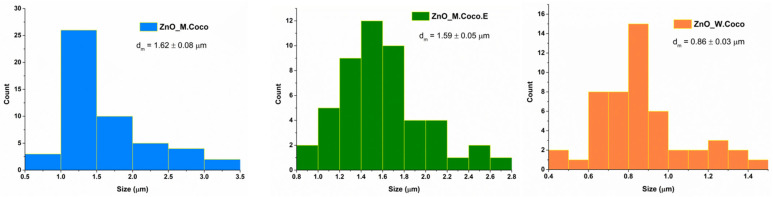
Size distribution histograms obtained for ZnO_M.Coco, ZnO_M.Coco.E, ZnO_W.Coco after thermal treatment at 800 °C for 2 h.

Regarding the particle measurements after the thermal treatment at 800 °C (Figure 8), they were easier to perform than those for the samples treated at 400 °C. This was due to the well-defined morphologies observed for the ZnO_M.Coco and ZnO_M.Coco.E samples. Meanwhile, the measurements were slightly more challenging for ZnO_W.Coco sample. The grain size distribution obtained after the sintering process indicates values ranging from 0.5 to 3.5 µm for ZnO_M.Coco, from 0.8 to 2.8 µm for ZnO_M.Coco.E, and from 0.4 to 1.5 µm for ZnO_W.Coco.

The XRD results confirm the formation of ZnO in all three Zn-based powders, and the white coloration of the powders is successfully achieved. However, despite the favorable phase composition, the powders’ sintering is a drawback for their intended application in wound dressing development. In addition, the ZnP phase appears well established, as it remains detectable in the diffraction patterns even after thermal treatment at 800 °C. Therefore, an alternative thermal treatment was selected for the three Zn-based powders, involving a temperature below 800 °C but above 400 °C, to balance phase formation while limiting excessive sintering.

### 2.3. Results of the Characterization of Zinc-Based Powders (ZnO_M.Coco, ZnO_M.Coco.E, and ZnO_W.Coco) After Thermal Treatment at 550 °C for 2 h, Followed by 650 °C for 2 h

**Figure 9:**
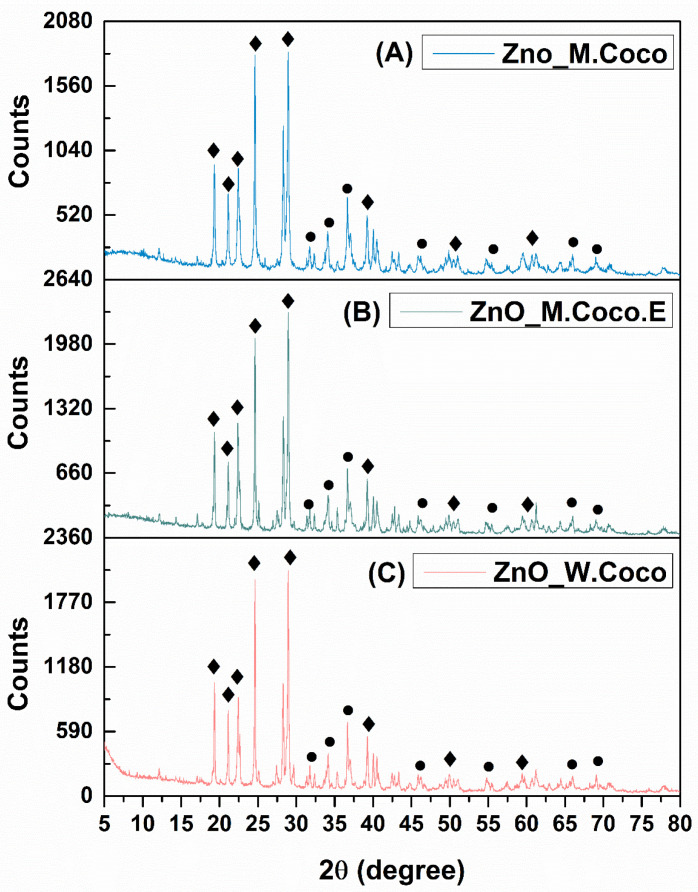
XRD diffraction patterns obtained for ZnO_M.Coco (**A**), ZnO_M.Coco.E (**B**) and ZnO_W.Coco (**C**) after thermal treatment at 550 °C for 2 h, followed by another thermal treatment at 650 °C for 2 h. The diffraction peaks corresponding to ZnO are marked with circles (●), while the peaks associated with Zn_3_(PO_4_)_2_ are indicated with diamonds (♦).

Given that the 800 °C, 2 h thermal treatment yielded positive results for crystallinity but negative effects on particle morphology, the next experimental step was to apply a lower temperature, 550 °C, for 2 h. After this step, the powders exhibited a black coloration, similar to that observed after thermal treatment at 400 °C for 3 h. Consequently, an additional thermal treatment at 650 °C for 2 h was applied. Following this two-step thermal treatment, the powders exhibited different visual appearances: a dark gray color for the ZnO_M.Coco sample; a light gray color for the ZnO_M.Coco.E sample; a black color for the ZnO_W.Coco sample. Figure 9A presents the X-ray diffraction pattern obtained for the ZnO_M.Coco powder after thermal treatment at 550 °C for 2 h, followed by an additional 2 h at 650 °C.

The diffraction pattern highlights crystalline features similar to those observed for the sample after thermal treatment at 800 °C for 2 h, in terms of peak identification and phase composition. However, the diffraction peaks broaden. This result indicates the formation of crystalline ZnO and Zn_3_(PO_4_)_2_ phases. Similar crystalline characteristics are also observed in the diffraction pattern shown in Figure 9B, obtained for the ZnO_M.Coco.E sample after thermal treatment at 550–650 °C. Accordingly, a comparison between the diffraction patterns recorded at 800 °C and 550–650 °C reveals the presence of the same characteristic peak positions associated with the ZnO and Zn_3_(PO_4_)_2_ phases. However, in the sample treated at 550–650 °C, the previously observed diffraction peaks at approximately 21° 2θ are no longer detected. The XRD results for the ZnO_W.Coco samples are presented in Figure 9C. The selection of the thermal treatment temperatures and the total treatment duration of 550–650 °C for 4 h proved appropriate also for ZnO_W.Coco sample, whose diffraction pattern is very similar to that of the ZnO_M.Coco sample. This behavior correlates with the lower fat content of both coconut water and centrifuged coconut milk.

**Figure 10:**
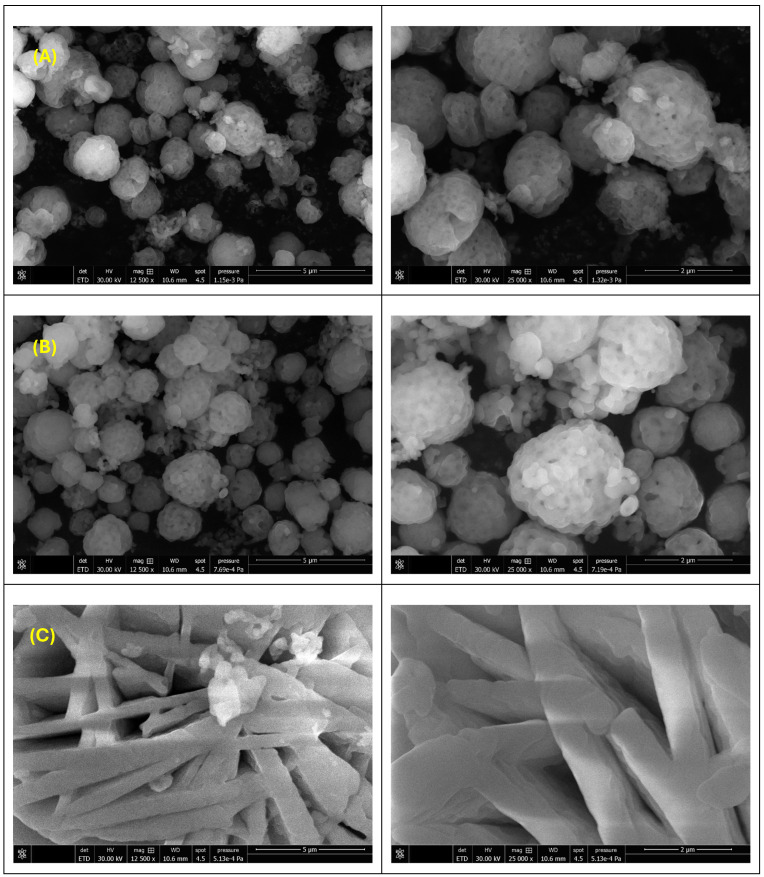
SEM micrographs obtained for ZnO_M.Coco (**A**), ZnO_M.Coco.E (**B**), ZnO_W.Coco (**C**) after thermal treatment at 550 °C for 2 h, followed by an additional treatment at 650 °C for 2 h.

The particle morphologies obtained after the two-step thermal treatment at 550–650 °C for a total duration of 4 h reveal trends similar to those observed for the samples treated at 800 °C for 2 h, namely a pronounced similarity between the powders synthesized using coconut milk and a substantial morphological difference compared to the sample synthesized using coconut water. Accordingly, the SEM micrographs presented in Figure 10A and Figure 10B correspond to the ZnO_M.Coco and ZnO_M.Coco.E samples, respectively. In both cases, small primary quasi-spherical particles with nanometric dimensions can be identified. These primary particles assemble into larger agglomerates with quasi-spherical morphologies and micron-scale sizes. The assembly of these nanosized particles results in porous agglomerates. In the case of the ZnO_M.Coco.E sample, the identification of the primary nanosized particles is more evident compared to the ZnO_M.Coco sample, where the individual particles are more difficult to distinguish. This behavior can be attributed to the residual organic phases originating from the green synthesis source, which are more pronounced in the non-centrifuged coconut milk and may promote partial particle bridging. In contrast, the ZnO_W.Coco sample synthesized using coconut water exhibits a completely different morphology. As shown in Figure 10C, elongated platelet-like structures with sizes ranging from the nanoscale to the microscale are observed. These platelets appear fused and randomly arranged, forming irregular assemblies without a clearly defined agglomerate structure.

**Figure 11:**
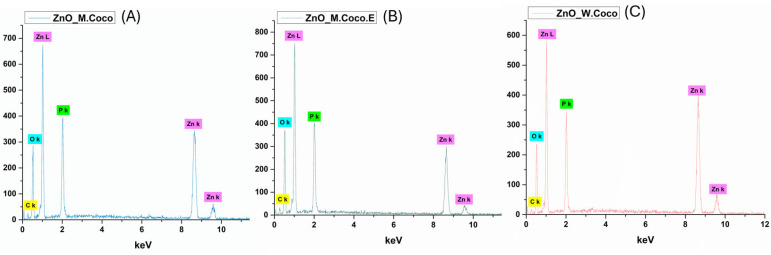
EDS results obtained for ZnO_M.Coco (**A**), ZnO_M.Coco.E (**B**), ZnO_W.Coco (**C**) after thermal treatment at 550 °C for 2 h followed by an additional treatment at 650 °C for 2 h.

The effect of the selected two-step thermal treatment at 550 °C for 2 h, followed by an additional treatment at 650 °C for 2 h, was also reflected in the elemental composition of the obtained powders. The EDS spectra presented in Figure 11 for ZnO_M.Coco (A), ZnO_M.Coco.E (B), and ZnO_W.Coco (C) clearly confirms the presence of Zn, O, and P as the main constituent elements. Compared to the samples treated at 400 °C, a substantial reduction in C content is observed (Table 3), which can be attributed to the more efficient removal of organic residues originating from the green synthesis sources. Specifically, the C content decreases to 7.70 wt% for ZnO_M.Coco, while for ZnO_M.Coco.E and ZnO_W.Coco, it is reduced to approximately 3 wt%. Concomitantly, increased amounts of Zn, O, and P are detected, consistent with the XRD results, further supporting the successful formation and stabilization of the two Zn-based phases, namely ZnO and ZnP.

**Figure 12:**
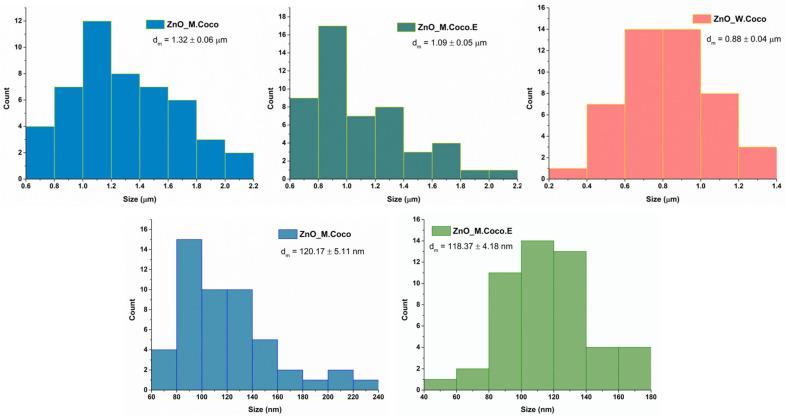
Size distribution histograms obtained for ZnO_M.Coco, ZnO_M.Coco.E, ZnO_W.Coco after thermal treatment at 550 °C for 2 h, followed by an additional treatment at 650 °C for 2 h. The measurements were performed on large quasi-spherical agglomerates observed for ZnO_M.Coco and ZnO_M.Coco.E samples, and on elongated platelet-like particles characteristic of the ZnO_W.Coco sample. Additional measurements were carried out on the small quasi-spherical primary particles forming the large agglomerates in ZnO_M.Coco and ZnO_M.Coco.E.

The particle size distribution histograms for the powders thermally treated at 550–650 °C were obtained from measurements performed on the large quasi-spherical agglomerates observed in the ZnO_M.Coco and ZnO_M.Coco.E samples, as well as on the platelet-like structures characteristic of the ZnO_W.Coco sample (Figure 12). For samples synthesized using coconut milk, relatively similar results were obtained, consistent with the morphological similarities observed between the two materials. The agglomerate sizes range between 0.6 and 2.2 µm for both samples, with average sizes of 1.32 ± 0.06 µm for ZnO_M.Coco and 1.09 ± 0.05 µm for ZnO_M.Coco.E. In contrast, the ZnO_W.Coco sample exhibits smaller structures, with sizes ranging between 0.2 and 1.4 µm and an average size of 0.88 ± 0.04 µm. In addition, measurements were also performed on the small particles that assemble into the large quasi-spherical agglomerates observed in the ZnO_M.Coco and ZnO_M.Coco.E samples. For ZnO_M.Coco, particle sizes ranged between 60 and 240 nm, with an average size of 120.17 ± 5.11 nm. Meanwhile, for ZnO_M.Coco.E, the particle sizes ranged between 40 and 180 nm, with an average value of 118.37 ± 4.18 nm.

Thus, considering all the results obtained following the three thermal treatments applied to the three Zn-based powders (ZnO_M.Coco, ZnO_M.Coco.E, and ZnO_W.Coco), it was demonstrated that the use of green synthesis sources based on coconut milk and coconut water leads not only to the formation of ZnO, but also to the formation of ZnP. Complete removal of organic residues derived from the green synthesis sources is achieved at 800 °C. However, under these conditions, the resulting particle morphology is not suitable for wound dressing development due to extensive grain growth and sintering. In contrast, the two-step thermal treatment at 550 °C followed by 650 °C represents an optimal compromise, enabling effective elimination of organic residues while preserving a favorable particle morphology and stable crystalline phases. Based on this balance between phase composition, morphological features, and overall physicochemical performance, the ZnO_M.Coco.E sample obtained after the 550–650 °C thermal treatment was selected in the design of hydrogel-based wound dressings.

### 2.4. Results of the Bioactive Wound Dressings Characterization

**Figure 13:**
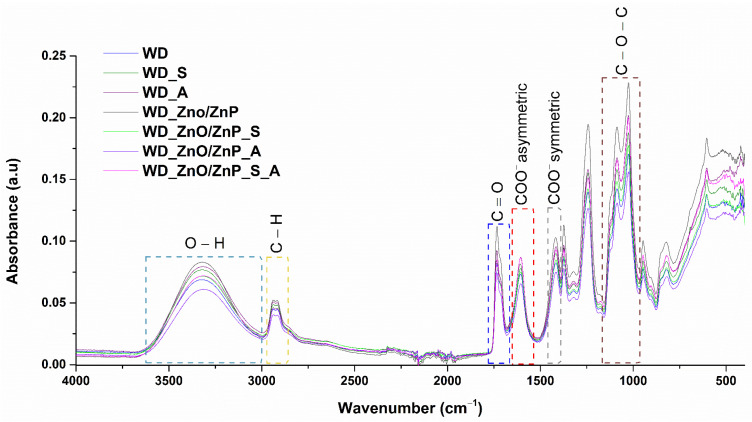
FTIR spectra recorded for all wound dressing formulations (WD, WD_S, WD_A, WD_ZnO/ZnP, WD_ZnO/ZnP_S, WD_ZnO/ZnP_A, WD_ZnO/ZnP_S_A).

The bioactive wound dressings were analyzed by FTIR spectroscopy, and the resulting spectra are shown in Figure 13. The recorded spectra for all formulations (WD, WD_S, WD_A, WD_ZnO/ZnP, WD_ZnO/ZnP_S, WD_ZnO/ZnP_A, WD_ZnO/ZnP_S_A) clearly exhibit similar absorption band patterns, confirming the preservation of the primary polymeric structure across all samples. However, slight differences in band positions and intensities are observed, as detailed in Table 4, which summarizes the specific wavenumbers identified in each spectrum. The broad absorption band in the 3320–3330 cm^−1^ region is characteristic of O–H stretching modes originating from the hydroxyl groups present in all components of the wound dressing formulation, including PVA, ALG, and the incorporated bioactive compounds. The distinct band with two maxima between 2915 cm^−1^ and 2938 cm^−1^ corresponds to C–H stretching vibrations of aliphatic –CH_2_– groups originating from PVA and ALG, with additional contributions from the organic phytochemical constituents [59,60,61,62,63]. The spectral band at approximately 1734 cm^−1^ can be attributed to C=O stretching vibrations, likely associated with residual acetate groups in partially hydrolyzed PVA [64]. Additionally, this band may also reflect interactions between the polymer matrix and carbonyl-containing phytochemicals, particularly in formulations with aronia powder. ALG functional groups are evidenced by the presence of the COO^−^ asymmetric stretching vibration at around 1606 cm^−1^ and the symmetric stretching vibration at approximately 1415 cm^−1^. The band near 1415 cm^−1^ may also include contributions from C–H bending vibrations of PVA. The spectral region between 1025 cm^−1^ and 1085 cm^−1^ is attributed to C–O and C–O–C stretching vibrations characteristic of both constituent polymers, PVA and ALG. This region is dominated by polymer backbone vibrations but may also overlap with absorption bands associated with phytochemical components, particularly polysaccharides and phenolic compounds present in spirulina and aronia [65,66,67].

**Figure 14:**
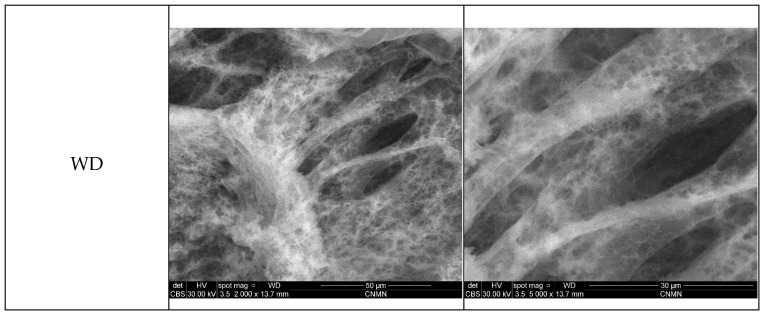

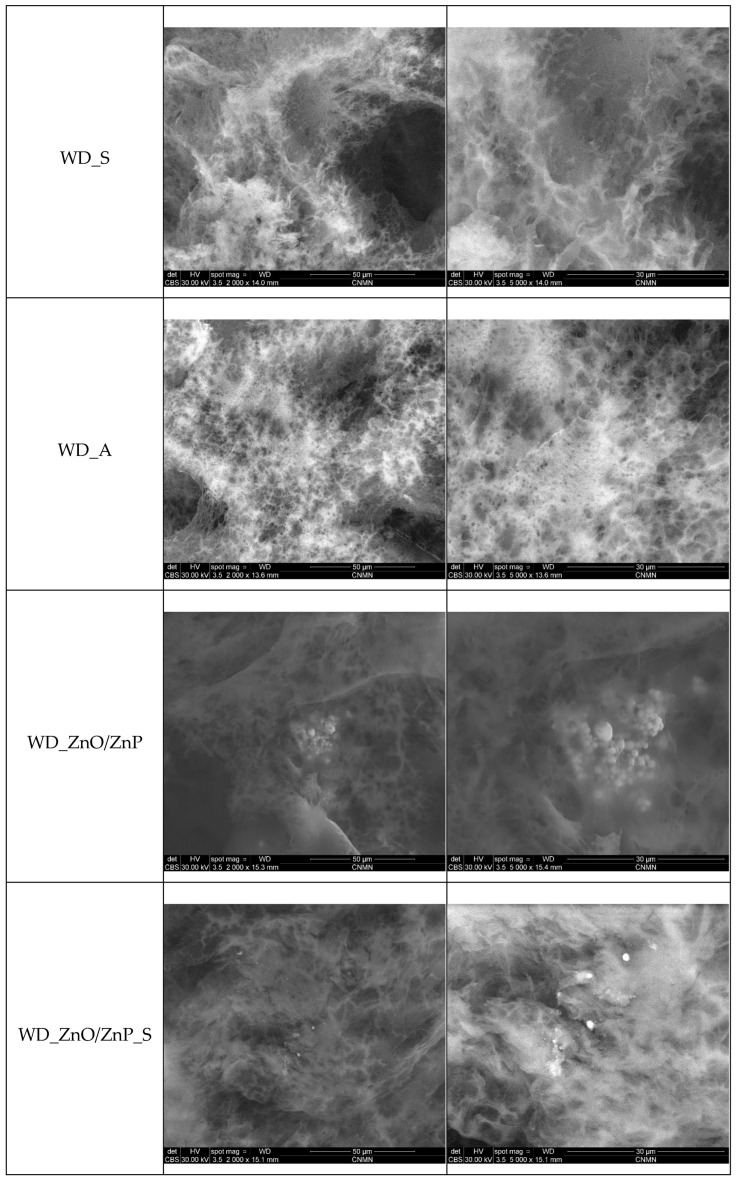

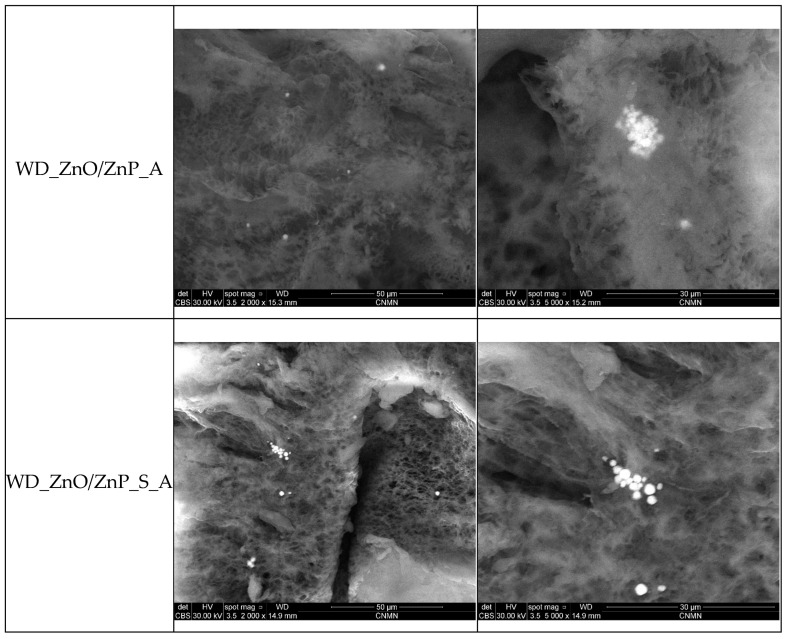
SEM micrographs performed at 2000× and 5000× for all wound dressing formulations (WD, WD_S, WD_A, WD_ZnO/ZnP, WD_ZnO/ZnP_S, WD_ZnO/ZnP_A, WD_ZnO/ZnP_S_A).

The SEM results are presented below and illustrated in Figure 14, showing representative micrographs recorded at magnifications of 2000× and 5000× for each formulated wound dressing.

The micrographs at 2000× magnification reveal the overall morphology of the wound dressings and the structural modifications induced by each bioactive addition. The control sample (WD) exhibits a morphology characteristic of porous hydrogels, with small pores distributed across the polymer sheets forming the aerated network of the dressing, as well as larger pores generated by the spatial arrangement and stacking of these polymer layers. Upon incorporation of spirulina powder (WD_S), the aerated structure of the polymer sheets is reduced, resulting in a more compact architecture. Nevertheless, the hierarchical pore structure is preserved, with larger pores still evident and smaller pores remaining, although partially filled or less open than in the control sample. Similarly, the addition of aronia powder (WD_A) induces morphological changes relative to WD, primarily through a decrease in sheet aeration. However, numerous small pores are identified, which appear more clearly defined compared to the control, while the larger pores formed through polymer sheet assembly are also maintained. In the case of formulations containing Zn-based nanoparticles, the general morphology of the wound dressing remains comparable to that of the control sample. A similar trend is observed for the spirulina-containing (WD_ZnO/ZnP_S) and aronia-containing (WD_ZnO/ZnP_A) composites, where the structural features resemble those of WD_S and WD_A, respectively. The formulation incorporating both botanical powders and Zn-based nanoparticles (WD_ZnO/ZnP_S_A) exhibits a combined morphology that reflects the structural characteristics of each additive, resulting in a more complex architecture. Distinct features are clearly visible in both the 2000× and 5000× micrographs of Zn-containing formulations, indicating the presence of Zn-based nanoparticles. These nanoparticles are identified as discrete spheres dispersed among the polymer sheets, distributed throughout the entire structure, either individually or forming agglomerates. Importantly, their distribution and morphological behavior appear consistent across all Zn-containing formulations, independent of the presence or absence of botanical additives. Therefore, the Zn-based nanoparticles are visibly embedded within the wound dressing matrix without significantly altering the overall porous morphology, indicating a uniform and homogeneous dispersion within the polymer network.

**Figure 15:**
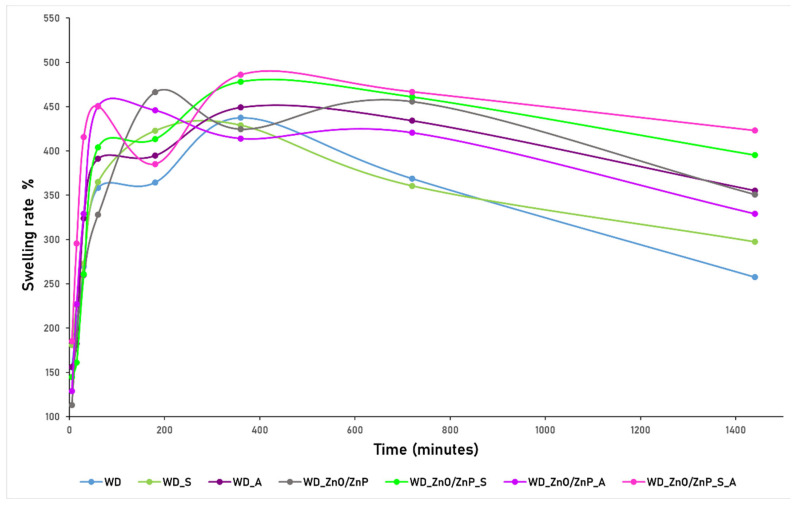
The swelling behavior over time of all wound dressing formulations (WD, WD_S, WD_A, WD_ZnO/ZnP, WD_ZnO/ZnP_S, WD_ZnO/ZnP_A, WD_ZnO/ZnP_S_A).

The next step involved evaluating the liquid-absorption behavior of the wound dressings over time to estimate their performance under conditions simulating contact with wound exudate. The dressings were monitored over a 24-h period (1440 min), and the results obtained are graphically presented in Figure 15.

**Figure 16:**
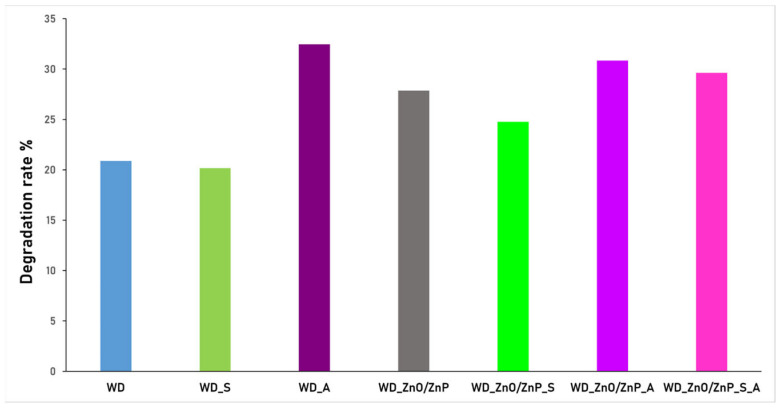
Degradation rate of all wound dressing formulations (WD, WD_S, WD_A, WD_ZnO/ZnP, WD_ZnO/ZnP_S, WD_ZnO/ZnP_A, WD_ZnO/ZnP_S_A) after 24 h of liquid immersion.

The overall swelling behavior of the formulated dressings is characteristic of hydrogel systems, showing a rapid increase in swelling rate during the first minutes of contact with the liquid medium, followed by a temporary equilibrium phase and, after 24 h, a gradual decrease in swelling capacity. Up to 60 min, all dressings exhibit a similar increasing profile. At 180 min, except for WD_S and WD_ZnO/ZnP, all other formulations show a slight decrease in swelling rate, followed by a renewed increase at 360 min, indicating structural rearrangement and further fluid uptake within the polymeric network. Comparative analysis reveals that the highest swelling performance is recorded for the complex formulation WD_ZnO/ZnP_S_A, followed in descending order by WD_ZnO/ZnP_S, WD_ZnO/ZnP, and WD_ZnO/ZnP_A. Meanwhile, the samples without Zn-based nanoparticles display comparatively lower swelling values. After 1440 min (24 h), a decrease in the swelling rate is observed for all formulations, which correlates with the onset of material degradation. This degradation behavior was evaluated after 24 h, and the corresponding results are presented graphically in Figure 16.

The degradation rate of the dressings after 24 h of immersion in the liquid medium ranged between 20% and 35%. The highest degradation was recorded for the WD_A sample, followed in descending order by WD_ZnO/ZnP_A, WD_ZnO/ZnP_S_A, WD_ZnO/ZnP, WD_ZnO/ZnP_S, WD_S, and finally the control sample WD. These results indicate that formulations containing aronia powder exhibit faster degradation than the other dressings. This effect may be associated with the physicochemical characteristics of the aronia powder, which is derived from dried fruit material and is generally less homogeneous and less finely dispersed than spirulina powder, obtained from microalgal biomass. Moreover, dressing morphology can influence the structural integrity of the hydrogel network and promote a higher degradation rate during prolonged immersion.

**Figure 17:**
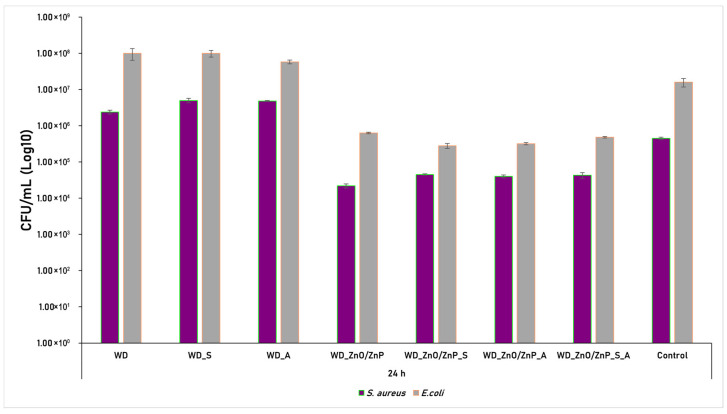
Results of the biofilm inhibition assessment expressed as log_10_ CFU/mL, performed on the Gram-positive bacterium *S.aureus* and the Gram-negative bacterium *E.coli* after 24 h of incubation with the wound dressing formulations (WD, WD_S, WD_A, WD_ZnO/ZnP, WD_ZnO/ZnP_S, WD_ZnO/ZnP_A, WD_ZnO/ZnP_S_A). The results are expressed as mean ± standard deviation (SD).

To investigate the biological interaction characteristics of the wound dressing formulations, particularly with respect to the antimicrobial efficacy of the Zn-based nanoparticles and the botanical powders of spirulina and aronia, biofilm modulation assays were performed. The ability of the formulations to influence monospecific biofilm formation was evaluated using two bacterial strains, *S. aureus* and *E. coli*, after incubation for 24 and 48 h. The results obtained for both strains after 24 h are presented in Figure 17.

The graphical representation of results for both *S. aureus* and *E. coli* after 24 h of incubation in the presence of wound dressing formulations highlights differences in antibacterial activity upon incorporation of Zn-based nanoparticles. Compared to the bacterial control (untreated cells incubated under the same conditions), the formulations WD, WD_S, and WD_A did not show a significant reduction in biofilm formation by *S. aureus* and *E. coli.* This suggests that the botanical powders alone do not exhibit pronounced antimicrobial activity under the tested conditions, or that their concentration may be insufficient to induce a measurable inhibitory effect. In contrast, the incorporation of Zn-based nanoparticles resulted in a significant decrease in biofilm formation for both *S. aureus* and *E. coli* relative to the bacterial control. The strongest inhibitory effect against *S. aureus* was observed for the WD_ZnO/ZnP formulation. In the case of *E. coli*, a moderate additional reduction in biofilm development was observed with the formulations containing botanical powders (spirulina, aronia, and their combination) compared with the Zn-based nanoparticle formulation alone, suggesting a potential contributory or synergistic role for the botanical components in modulating Gram-negative biofilm formation.

**Figure 18:**
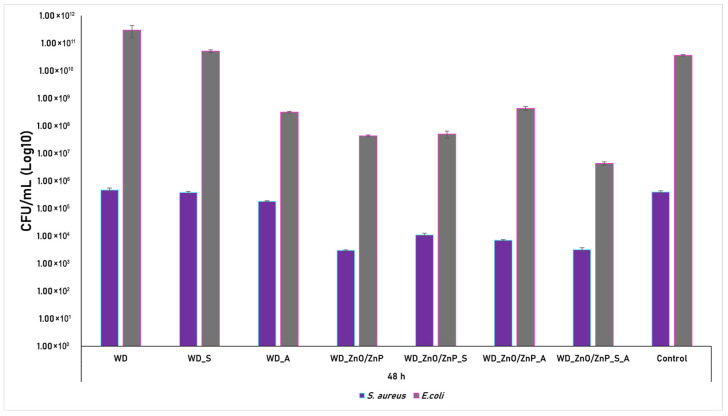
Results of the biofilm inhibition assessment expressed as log_10_ CFU/mL, performed on the Gram-positive bacterium *S.aureus* and the Gram-negative bacterium *E.coli* after 48 h of incubation with the wound dressing formulations (WD, WD_S, WD_A, WD_ZnO/ZnP, WD_ZnO/ZnP_S, WD_ZnO/ZnP_A, WD_ZnO/ZnP_S_A). The results are expressed as mean ± standard deviation (SD).

The effect was also evaluated after 48 h of incubation, and the results are presented in Figure 18.

After 48 h of incubation, a more pronounced antimicrobial trend associated with the botanical powders becomes evident. In the *S. aureus* strain, the aronia-containing dressing (WD_A) reduced biofilm formation compared to the bacterial control. The WD_ZnO/ZnP formulation demonstrated enhanced inhibitory activity at the extended time point, indicating a sustained antimicrobial effect. Moreover, the composite dressing containing both Zn-based nanoparticles and the botanical mixture (WD_ZnO/ZnP_S_A) showed an efficacy comparable to WD_ZnO/ZnP, while the formulations WD_ZnO/ZnP_S and WD_ZnO/ZnP_A also displayed significant reductions in biofilm formation compared to the bacterial control and relative to their respective 24-h results. In the case of *E. coli*, the 48-h results reveal a similar behavior for WD_A as observed for *S. aureus*, with a noticeable decrease in biofilm formation. The dressings containing Zn-based nanoparticles continued to exhibit a clear inhibitory effect against *E. coli* biofilm development, with the most pronounced activity recorded for WD_ZnO/ZnP_S_A. Overall, these findings suggest that prolonged interaction time enhances the antimicrobial performance of both Zn-based nanoparticles and botanical powders, supporting their synergistic contribution to biofilm inhibition.

**Figure 19:**
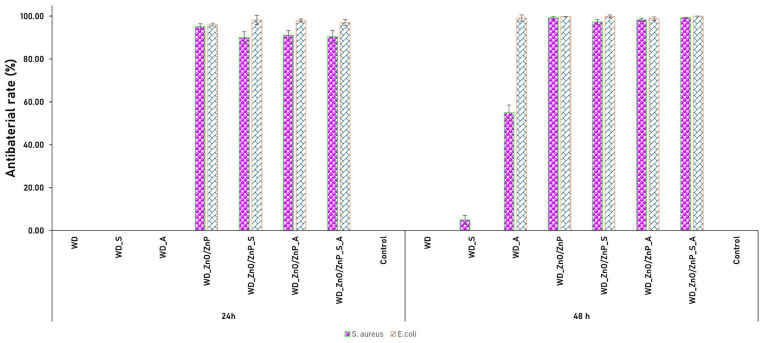
Antibacterial inhibition rate (%) of the wound dressing formulations (WD, WD_S, WD_A, WD_ZnO/ZnP, WD_ZnO/ZnP_S, WD_ZnO/ZnP_A, WD_ZnO/ZnP_S_A) against *S. aureus* and *E. coli* after 24 h and 48 h. Negative values (CFUsample > CFUcontrol) and CFUcontrol were displayed as 0%, indicating no antibacterial effect. The results are expressed as mean ± standard deviation (SD).

The antimicrobial activity of the samples was further quantified by calculating the antibacterial inhibition rate (%), as shown in Figure 19. The data confirm the limited activity of the WD, WD_S, and WD_A formulations, which, in some cases, even promoted bacterial growth. In contrast, the formulations containing Zn-based nanoparticles exhibited significant antibacterial activity, achieving high inhibition efficiency (>90%) after 24 h, which further increased to> 95% at 48 h against both tested strains, *S. aureus* and *E. coli*.

The biological performance of the developed wound dressings was evaluated by assessing multiple cellular aspects, including keratinocyte metabolic activity and membrane integrity, and macrophage nitric oxide production, to highlight the wound dressing materials’ biocompatibility and pro-inflammatory potential.

**Figure 20:**
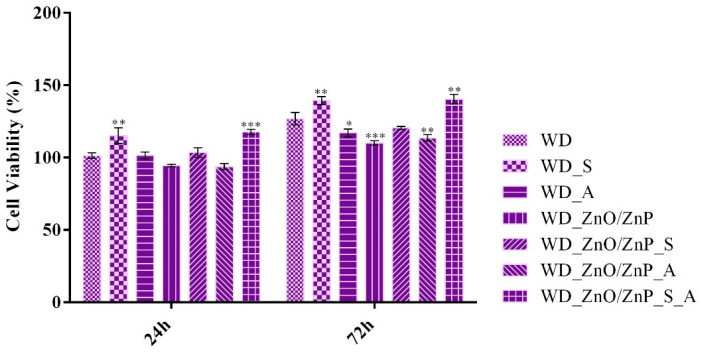
Graphical representation of human keratinocytes viability after 24 h and 72 h of culture in contact with the developed wound dressings (WD, WD_S, WD_A, WD_ZnO/ZnP, WD_ZnO/ZnP_S, WD_ZnO/ZnP_A, WD_ZnO/ZnP_S_A). The experimental control was represented by a simple PVA/ALG wound dressing (WD), which was defined as 100% cell viability at 24 h. The results are presented as mean ± standard deviation (SD). Statistical analysis was performed using GraphPad Prism version 9, with two-way ANOVA followed by Bonferroni’s post hoc test, and statistical significance set at *p* ≤ 0.05. Significance levels compared to the control group are indicated as follows: *p* ≤ 0.05 (*), *p* ≤ 0.01 (**), and *p* ≤ 0.001 (***).

Cell viability of human keratinocytes was evaluated by measuring metabolic activity at 24 h and 72 h after exposure to the developed wound dressings (Figure 20). After 24 h, most formulations showed cell viability comparable to that of the simple WD (control), indicating good cytocompatibility after short-term exposure. A significant increase in metabolic activity was observed in the spirulina-based formulation (WD_S) compared with the control, suggesting that the natural compound has a stimulatory effect on keratinocyte proliferation. In contrast, HaCaT cells cultured on WD_ZnO/ZnP showed decreased cell viability compared with the control. However, this decrease was not significant and remained above the cytotoxicity threshold, indicating that incorporating the particles into the wound dressing structure does not induce a significant cytotoxic effect. Moreover, the addition of bioactive compounds to the ZnO/ZnP dressing improved cell viability, which was similar to that of the experimental control. Notably, the combined formulation (WD_ZnO/ZnP_S_A) led to a significant increase in metabolic activity compared with simple WD, suggesting that incorporating both bioactive compounds is an effective strategy to mitigate potential nanoparticle-associated cytotoxicity while enhancing keratinocyte proliferation. At 72 h, most WD formulations exhibited metabolic activity similar to the experimental control, confirming cytocompatibility during prolonged exposure. The ZnO/ZnP-based dressing still showed significantly lower viability than the control, whereas incorporating spirulina or aronia into the nanoparticle matrix restored cell viability to levels similar to the WD baseline, suggesting that bioactive compounds attenuate nanoparticle-associated cellular stress. The combined system (WD_ZnO/ZnP_S_A) presented a significant increase in cell viability, indicating that the bioactive synergic effects stimulate and sustain keratinocyte proliferation. Moreover, comparing 72 h to 24 h data showed an overall increase in cell viability across all samples, indicating that all formulations enhance cell proliferation.

**Figure 21:**
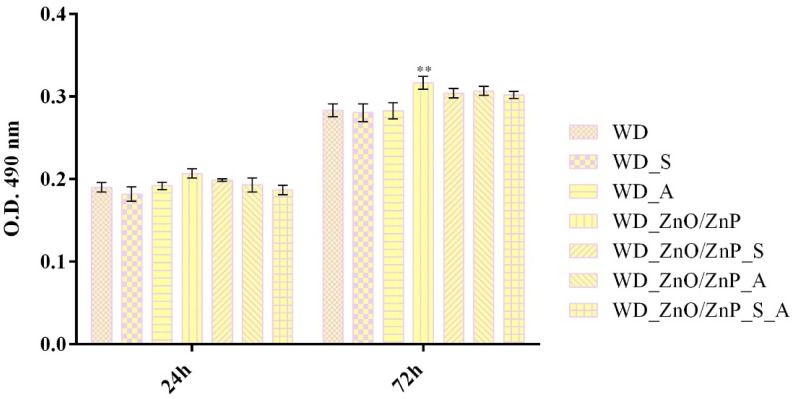
Graphical representation of LDH levels in human keratinocyte cultures released after 24 h and 72 h of culture incubated with the developed wound dressings (WD, WD_S, WD_A, WD_ZnO/ZnP, WD_ZnO/ZnP_S, WD_ZnO/ZnP_A, WD_ZnO/ZnP_S_A). LDH levels were expressed relative to the experimental control (simple WD). The results are presented as mean ± standard deviation (SD). Statistical analysis was performed using GraphPad Prism version 9, with two-way ANOVA followed by Bonferroni’s post hoc test, and statistical significance set at *p* ≤ 0.05. Statistical significance compared with the control group is indicated as *p* ≤ 0.01 (**).

To further investigate the cytotoxic potential of the developed wound dressings, the LDH assay was employed to measure LDH release at 24 h and 72 h after cell-material interaction (Figure 21). At 24 h, LDH levels for all formulations were comparable to those of the simple WD control, indicating that short-term exposure did not compromise membrane integrity. At 72 h, LDH values for most samples remained similar to WD, suggesting that prolonged contact did not lead to progressive membrane damage in human keratinocytes. The ZnO/ZnP-containing formulation showed a statistically considerable increase in LDH release compared with simple WD, suggesting a moderate effect on membrane integrity. In contrast, incorporating bioactive compounds (spirulina and/or aronia) into the WD_ZnO/ZnP did not significantly alter LDH levels compared with the control. These findings suggest that the addition of natural bioactive components attenuates the membrane damage associated with ZnO/ZnP incorporation. Taken together with the MTT results, the LDH data confirm that the developed wound dressings do not induce relevant cytotoxic membrane damage and exhibit an overall favorable cytocompatibility profile.

**Figure 22:**
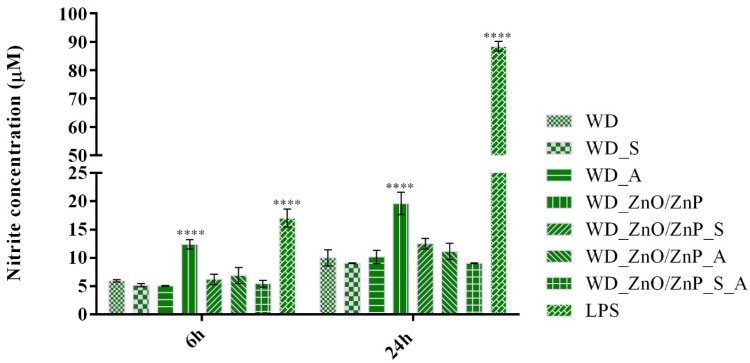
Graphical representation of nitric oxide (NO) production by RAW 264.7 after 6 h and 24 h of direct contact with the developed wound dressings. Nitrite accumulation in the culture supernatant was quantified using the Griess reaction and expressed as µM nitrite. The simple WD served as the experimental control. Lipopolysaccharide (LPS) was used as a positive control. The results are presented as mean ± standard deviation (SD). Statistical analysis was performed using GraphPad Prism version 9, with two-way ANOVA followed by Bonferroni’s post hoc test, and statistical significance set at *p* ≤ 0.05. Statistical significance compared with the LPS group is indicated as *p* ≤ 0.0001 (****).

Nitric oxide (NO) production was quantified in RAW 264.7 macrophages after 6 h and 24 h of direct contact with the developed wound dressings as an indicator of pro-inflammatory potential (Figure 22). At 6 h, nitrite levels for most formulations were comparable to those of the simple WD control, indicating the absence of early macrophage activation. A statistically considerable increase in NO production was observed for the ZnO/ZnP-containing formulation relative to WD, suggesting moderate macrophage activation likely associated with nanoparticle incorporation. However, the levels were significantly lower than those observed for the LPS-stimulated positive control, indicating that the response reflects mild immunostimulatory activity rather than a pronounced inflammatory reaction. In contrast, the bioactive-enriched formulations (WD_ZnO/ZnP_S, WD_ZnO/ZnP_A, and WD_ZnO/ZnP_S_A) did not exhibit significant differences compared to the control. At 24 h, the trend was similar: the WD_ZnO/ZnP formulation continued to show significantly higher nitrite levels than WD, whereas the incorporation of spirulina and/or aronia resulted in NO values that were not statistically different from the control. These findings suggest that while Zn-based nanoparticles may induce moderate macrophage activation, the addition of natural bioactive compounds effectively modulates this response, maintaining NO production within a controlled range. Overall, NO levels for all experimental formulations remained markedly lower than those measured in the LPS-stimulated group, confirming the safety of the developed wound dressings.

## 3. Discussion

Coconut milk and coconut water are derived from the coconut fruit, produced by the coconut palm (*Cocos nucifera* L.), one of the most widely used trees worldwide. Coconut water naturally forms inside the coconut cavity and is surrounded by coconut meat, which is in turn enclosed by the hard shell and fibrous husk. To produce coconut milk, the coconut meat is mechanically pressed using various techniques and may be mixed or not with fresh water [68]. Given their biological origin, both coconut milk and coconut water contain essential minerals, including potassium, magnesium, phosphorus, and iron [69,70,71,72,73]. One of the earliest studies addressing the interaction between zinc ions and phosphorus was published in 1973 by Nriagu, who demonstrated that Zn ions are rapidly converted into hopeite, the hydrated form of ZnP with the chemical formula Zn_3_(PO_4_)_2_·4H_2_O, when present in dilute phosphate-containing solutions [56,74]. Thus, considering the synthesis conditions applied for the three powder type materials (ZnO_M.Coco, ZnO_M.Coco.E, ZnO_W.Coco), which involved the use of coconut milk and coconut water containing phosphorus in mineral form, together with a zinc nitrate hexahydrate solution supplying Zn^2+^ ions, the formation of two distinct crystalline phases was promoted. This outcome was clearly evidenced by the XRD results obtained after the first thermal treatment at 400 °C for 3 h, namely the formation of ZnO alongside the hydrated ZnP phase, hopeite (Zn_3_(PO_4_)_2_·4H_2_O). The formation of the hopeite phase is directly related to the relatively low calcination temperature of 400 °C. It is well established that at low calcination temperatures, typically between 200 °C and 240 °C, ZnP dihydrate (Zn_3_(PO_4_)_2_·2H_2_O) is formed, while temperatures up to 400 °C favor the formation of the tetrahydrated phase Zn_3_(PO_4_)_2_·4H_2_O. At higher calcination temperatures, the anhydrous ZnP phase (Zn_3_(PO_4_)_2_) is obtained, which becomes stable in the temperature range of approximately 600–650 °C, without further structural transformations [75,76]. Moreover, the XRD patterns recorded after thermal treatment at 400 °C exhibit a characteristic diffraction peak at approximately 9° 2θ, which correlates with the formation of orthorhombic hopeite (Zn_3_(PO_4_)_2_·4H_2_O), whose first intense reflection appears at this angle. In contrast, the diffraction patterns obtained after thermal treatments at 800 °C and 550–650 °C show no low-angle peak, with the first detectable reflection appearing at approximately 19° 2θ. This shift confirms the transformation toward the anhydrous ZnP phase (Zn_3_(PO_4_)_2_), which crystallizes in a monoclinic structure [77,78]. The formation of ZnP alongside ZnO highlights the inherent complexity of green synthesis routes based on biologically derived matrices. Such systems are prone to generating multifunctional Zn-based materials, rather than single-phase oxides, as a direct consequence of the rich and reactive mineral composition of natural synthesis sources.

With regard to the distinct particle morphologies obtained for the three types of powders, a direct correlation between a specific crystalline phase (ZnO or ZnP) and a particular morphology cannot be unequivocally established. However, according to literature data, ZnO typically exhibits rod-shaped particles, although spherical, flower-like, raspberry-like, acicular, or granular morphologies have also been widely reported [79]. In contrast, zinc phosphate (Zn_3_(PO_4_)_2_) is most frequently described as forming plate-like structures, due to the preferential growth directions induced by phosphate groups [80,81]. From a dimensional perspective, ZnO particles are commonly reported in both nanometric and micrometric ranges, whereas ZnP is more often associated with micrometric particles [82]. Temperature, calcination time, and reaction medium are also critical parameters that govern morphological evolution in ZnO and ZnP [83,84,85,86,87]. While clear morphological differences were observed between ZnO_M.Coco and ZnO_M.Coco.E after thermal treatment at 400 °C, their morphologies became considerably more similar after treatment at 800 °C and at 550–650 °C. In the case of ZnO_M.Coco, where non-centrifuged coconut milk rich in organic components was used, residual organics that do not completely decompose at lower temperatures may influence particle growth. Lipidic fractions can act as capping or templating agents during particle formation, promoting aggregation and affecting final morphology [88]. In contrast, lipid reduction via centrifugation may mitigate these effects. At 800 °C, a significant increase in particle size was observed, consistent with thermally induced grain growth and sintering phenomena.

The primary objective was to obtain ZnO nanoparticles via green synthesis methods, but due to interactions during the formation reaction, ZnP particles were also formed as a secondary phase. This outcome is particularly advantageous for the intended application of developing bioactive wound dressings with antimicrobial and wound-healing acceleration properties, as considerable attention has been devoted to ZnO nanoparticles in this field and, more recently, to ZnP particles [37,89,90,91]. The modification and enhancement of the dressings through the incorporation of botanical powders and Zn-based nanoparticles were evaluated, and evidence of physicochemical interactions within the formulations was obtained from FTIR and SEM analyses. In the FTIR spectra, no major structural differences were observed among the formulations. However, slight shifts in the positions of certain absorption bands indicate the presence of intermolecular interactions. For instance, in formulations containing botanical powders, a shift in the hydroxyl characteristic band (around 3000 cm^−1^) was observed. Specifically, the control sample (WD) exhibits an absorption band at 3323 cm^−1^, whereas in WD_A, WD_ZnO/ZnP_S, WD_ZnO/ZnP_A, and WD_ZnO/ZnP_S_A, the corresponding band appears at 3314 cm^−1^. This shift suggests enhanced interactions between the polymer matrix and aronia and/or the Zn-based powder, leading to a denser hydrogen-bonding network [92,93]. On the other hand, the characteristic bands of the botanical powders and those of the constituent polymers overlap significantly in terms of wavenumber, making their individual identification more challenging. Similarly, in the formulations containing aronia, a slight shift is observed from 822 cm^−1^ in the control sample (WD) to 819 cm^−1^ in WD_A and WD_ZnO/ZnP_A, and to 820 cm^−1^ in WD_ZnO/ZnP_S_A. This vibrational band can be attributed to out-of-plane deformation vibrations of unsaturated C–H bonds associated with aromatic structures present in the aronia powder [66]. Furthermore, the low concentration of ZnO and the high-component polymeric matrix do not allow for clear detection of Zn–O vibrations in the FTIR spectra of the formulations containing Zn-based nanoparticles [94,95]. Nevertheless, the presence of Zn-based nanoparticles in the developed formulations was confirmed by SEM, which revealed spherical particles uniformly distributed within the polymer layers and throughout the porous structure of the wound dressings. Moreover, the addition of spirulina and aronia powders may contribute to the formation and stabilization of the hydrogel network. Both botanical additives contain bioactive compounds such as polysaccharides, proteins, and polyphenols bearing hydroxyl and carboxyl functional groups, which can interact with the polymer chains through hydrogen bonding. These interactions may promote additional physical crosslinking within the formulations, contributing to the development of a more compact morphology. Furthermore, the botanical powders may act as natural stabilizing agents for the Zn-based nanoparticles, facilitating their dispersion within the hydrogel matrix and limiting excessive nanoparticle aggregation [96,97,98,99]. Regarding the swelling profile of the dressings, this behavior is characteristic of hydrogels. An initial rapid swelling stage can be identified, correlated with the hydrophilic nature of ALG and PVA and the polymer–water interactions. This initial process is governed by osmotic pressure, which supports water diffusion into the porous matrix and its retention within the polymer structure. Subsequently, at equilibrium, the crosslinked polymer network balances osmotic forces, leading to a maximum swelling point. After this stage, partial relaxation of the polymer network and the onset of material degradation occur, resulting in a reduction in water retention capacity [100,101,102,103].

The evaluation of the antimicrobial efficacy of the developed formulations demonstrated that Zn-based nanoparticles played a dominant role in inhibiting biofilm formation for both *S. aureus* and *E. coli*. These findings are consistent with the literature, which reports the well-documented antimicrobial activity of ZnO and Zn_3_(PO_4_)_2_ nanomaterials. The inhibitory effect is primarily attributed to the release of Zn^2+^ ions, which disrupt the bacterial cell membrane and generate oxidative stress. Together, these mechanisms impair bacterial viability and interfere with biofilm development [37,46,104,105,106,107]. As a secondary effect, antimicrobial activity was also observed for the botanical powders. Although no pronounced inhibitory response was evident after 24 h, a moderate antimicrobial contribution became apparent after 48 h of incubation. Differences were observed between the spirulina and aronia-containing dressings, likely due to their distinct phytochemical compositions. Aronia is rich in anthocyanins and phenolic acids, compounds known to destabilize bacterial membranes and interfere with cellular processes [50,51,52]. Spirulina contains phycocyanin and other bioactive metabolites that exhibit mild antimicrobial activity [47,48,49]. Nevertheless, it is widely recognized that the antibacterial mechanisms of such phytochemicals are generally more limited compared to those of Zn-based nanoparticles. The most pronounced antimicrobial effect was recorded for the WD_ZnO/ZnP_S_A formulation, which combines Zn-based nanoparticles with both botanical powders. This outcome suggests that the botanical constituents may enhance or support the activity of the inorganic phase. Overall, the antimicrobial performance of the developed dressing appears to rely on a combination of mechanisms, including Zn^2+^ ion release, oxidative stress induction, and membrane destabilization, with Zn-based nanoparticles acting as the primary active agents and botanical components contributing to the enhancement and prolongation of the antimicrobial effect. The mechanism of action of Zn^2+^ ions released from Zn-based nanoparticles has been extensively investigated, and clear interactions between these ions and bacterial cell membranes have been reported. Zn^2+^ ions can disrupt the integrity of the bacterial membrane, leading to the inhibition of metabolic activity. In addition, the presence of Zn-based nanoparticles may induce oxidative stress through the generation of reactive oxygen species (ROS), which trigger a cascade of processes that damage essential cellular components such as proteins, lipids, and nucleic acids [108,109].

The developed wound dressings demonstrated a generally favorable biological profile, as evidenced by in vitro assays performed on human keratinocytes and murine macrophages. Overall, the materials did not induce cytotoxic effects, supporting the intrinsic biocompatibility of the polymeric matrix composed of ALG and PVA. Both polymers have been extensively investigated and validated for wound-healing applications, and the present biological evaluation further confirms their suitability for such applications [23,110,111,112,113,114]. Although green synthesis routes offer clear sustainability advantages, they may also introduce certain compositional variations that can influence biological behavior [115,116]. In this context, a slight increase in LDH release and nitric oxide production was observed in formulations containing Zn-based nanoparticles, suggesting a mild cellular stress response. However, these effects remained within acceptable limits and were not associated with overt cytotoxicity. Notably, the incorporation of botanical powders (spirulina and aronia) appeared to attenuate the impact of Zn-based nanoparticles, contributing to a more balanced cellular response. While the botanical additives exhibited only mild direct antimicrobial effects, their primary contribution is likely to support physiological wound-healing processes through bioactive compounds with antioxidant and regenerative potential [117,118,119,120]. Collectively, the results indicate that the combined system achieves an effective balance between antimicrobial performance and cellular compatibility, which is essential for advanced wound dressing applications.

Considering all the aspects and results obtained in this study, the formation of two Zn-based phases through a green synthesis route using coconut-derived media may represent an additional element of novelty in this field of research. The natural presence of diluted mineral phosphorus in the synthesis medium promotes the formation of a mixed ZnO/Zn_3_(PO_4_)_2_ system, which can be further exploited in wound dressing formulations. The coexistence of these two Zn-based phases may provide complementary antimicrobial effects, thereby enhancing antibacterial performance. At the same time, the green approach was maintained by incorporating botanical powders known to support wound-healing processes. The functionality of the developed dressings arises from the combined activity of the components, including Zn-based nanoparticles, the polymeric hydrogel network, and the botanical additives. Overall, the use of a sustainable synthesis strategy, together with the formation of Zn-based nanoparticles and their integration within polymeric networks enriched with botanical powders, demonstrates the potential of the proposed formulations to support antimicrobial activity and promote skin regeneration.

## 4. Conclusions

In the present study, three green synthesis routes were employed to obtain Zn-based nanoparticles using coconut-derived media. Based on the physicochemical characteristics required for the intended application, one nanoparticle formulation was selected for incorporation into hydrogel wound dressings. To further enhance biocompatibility and therapeutic potential, spirulina and aronia powders were incorporated as botanical additives. The synergistic interaction between the polymeric matrix, green-synthesized Zn-based nanoparticles, and botanical components offers a multifunctional solution by integrating the individual benefits of each constituent. The resulting dressings exhibited heterogeneously distributed pores, with slight structural variations depending on the incorporated botanical powder, and a uniform distribution of Zn-based nanoparticles, occasionally forming aggregates. A characteristic hydrogel swelling pattern was observed, with a rapid swelling increase exceeding 100% during the initial minutes of immersion, followed by a transient equilibrium phase and the onset of degradation after 24 h. From an antimicrobial perspective, testing against Gram-positive *S. aureus* and Gram-negative *E. coli* confirmed the significant antibiofilm activity of Zn-based nanoparticles. On the other hand, the biological evaluation performed on human keratinocytes and murine macrophages highlighted the predominant contribution of spirulina and aronia within the developed formulations. While incorporating Zn-based nanoparticles induced mild variations in LDH release and NO production, adding botanical powders led to a more balanced interaction between the nanoparticles and the cellular models. Consequently, the combined system achieved enhanced biocompatibility while maintaining antimicrobial functionality, reinforcing its potential for applications in advanced wound healing.

## 5. Materials and Methods

To fabricate the hydrogel dressings, the first step involved the synthesis of zinc oxide (ZnO) nanoparticles. A green synthesis route was employed, using coconut milk (coded as M.Coco) and coconut water (coded as W.Coco) as reducing and stabilizing agents. The coconut milk was sourced from canned commercial products, while the coconut water was freshly extracted from a commercially available coconut. Both sources were purchased from a local supermarket. The ZnO precursor used was zinc nitrate hexahydrate (Zn(NO_3_)_2_·6H_2_O) purchased from Sigma-Aldrich, and ultrapure water was used as the solvent.

The materials employed in the preparation of the hydrogel matrices included sodium alginate (ALG) from Sigma-Aldrich (St. Louis, MO, USA) and polyvinyl alcohol (PVA) with a polymerization degree of approximately 2000 and a saponification degree of ~80 mol%, supplied by Tokyo Chemical Industry (TCI) (Tokyo, Japan). Two natural powders were also used: one derived from Spirulina and another from Aronia fruit, both acquired from a local supplier in Romania.

### 5.1. Synthesis of Zinc Oxide Nanoparticles

In this study, three distinct green synthesis routes were explored for obtaining zinc oxide nanoparticles. The aim was to identify the method that yields nanoparticles possessing the most suitable physicochemical properties for incorporation into hydrogel-based wound dressings. The following section provides a detailed description of the three synthesis approaches employed.

#### 5.1.1. Green Method 1—Coconut Milk

The first step involved measuring 100 mL of coconut milk, followed by heating on a hot plate at 70 °C for 15 min. Meanwhile, a 0.3 M precursor solution of (Zn(NO_3_)_2_·6H_2_O) was prepared in a total volume of 100 mL and kept under magnetic stirring. After the coconut milk reached the desired temperature, the precursor solution was slowly added dropwise while stirring continuously. The reaction system was maintained at 70 °C while stirring continuously for 2 h. To collect the resulting ZnO nanoparticles, the solution was loaded into 50 mL centrifuge tubes, followed by centrifugation at 6000 rpm for 10 min. The pellet was washed three times with ultrapure water and centrifuged again after each washing step. Finally, the recovered pellet was dried at 100 °C for 12 h in a laboratory oven. The resulting sample was coded as ZnO_M.Coco.

#### 5.1.2. Green Method 2—Centrifuged Coconut Milk (Extract)

Given the high fat content of coconut milk, 150 mL was centrifuged at 6000 rpm for 10 min. Following this step, a distinct separation of fat was observed as a thick layer at the surface of the centrifuge tubes. This fat layer was carefully removed, and the remaining liquid, designated as the extract (E), was used for the synthesis process. The synthesis procedure followed the same steps as described in Green Method 1, with the only difference being the use of centrifuged coconut milk rather than the raw form. The resulting sample was labeled as ZnO_M.Coco.E.

#### 5.1.3. Green Method 3—Coconut Water

Coconut water, like coconut milk, contains various bioactive compounds capable of reacting with the zinc precursor to form zinc oxide, but it lacks the lipid content present in coconut milk. For this method, coconut water was collected from a commercially available coconut and filtered through gauze to remove any impurities, resulting in a clear solution. The synthesis followed the same protocol, reagent volumes, and precursor concentration as described in Green Methods 1 and 2 to allow a comparative evaluation of the resulting materials. The final product was labeled as ZnO_W.Coco.

**Figure 23:**
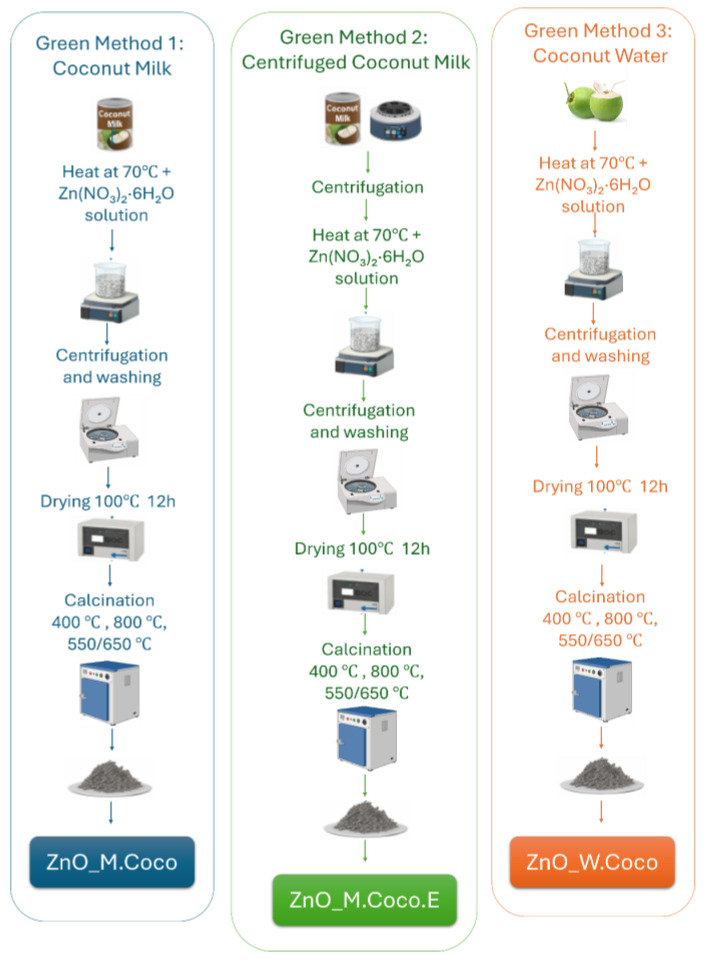
Schematic representation of zinc oxide green synthesis from coconut milk and water.

After drying all three samples at 100 °C for 12 h in a laboratory oven, a thermal calcination step was performed to eliminate residual organic components from coconut milk and water and to promote the formation of crystalline zinc oxide. The thermal treatment was performed using a Carbolite CWF1200 muffle furnace with a controlled heating rate of 20 °C min^−1^. Three different calcination protocols were applied: (1) 400 °C for 3 h, (2) 800 °C for 2 h, and (3) a sequential treatment involving 550 °C for 2 h followed by 650 °C for an additional 2 h. The synthesis methods and corresponding sample codes are schematically illustrated in Figure 23 and detailed in Table 5**.**

### 5.2. Preparation of PVA/ALG-Based Hydrogel Wound Dressings

Poly(vinyl alcohol) (PVA) and sodium alginate (ALG) were selected as polymer matrices for the preparation of hydrogel wound dressings. Aqueous stock solutions of PVA (10% *w*/*v*) and ALG (5% *w*/*v*) were prepared separately and subsequently mixed at a 50:50 volumetric ratio (*v*/*v*) to obtain all hydrogel formulations. A total of seven hydrogel formulations were prepared, all sharing the same polymer composition and concentrations, differing only in the type and amount of incorporated bioactive agents. The formulation without bioactive additives served as the control and was denoted WD (wound dressing).

Hydrogels containing individual botanical bioactive agents were first prepared. Spirulina powder and aronia powder were independently incorporated into the polymeric matrix at a concentration of 0.1 wt% relative to the total dry polymer mass, yielding the formulations denoted as WD_S and WD_A, respectively.

In addition, a zinc-loaded hydrogel was developed by incorporating the nanoparticles synthesized via a green route. Based on the physicochemical characterization results of the green-synthesized Zn-based samples, a single formulation (ZnO_M.Coco.E) was selected for hydrogel fabrication. The ZnO_M.Coco.E powder was added to the polymer mixture at a concentration of 0.2 wt% relative to the total dry polymer mass. The resulting hydrogel was denoted as WD_ZnO/ZnP, reflecting the two-phase composition identified by XRD analysis, which consisted of ZnO together with zinc phosphate (Zn_3_(PO_4_)_2_), referred to as ZnP.

Hybrid hydrogel formulations combining ZnO/ZnP nanoparticles with botanical additives were also prepared. One formulation incorporated spirulina powder (0.1 wt%) together with ZnO_M.Coco.E nanoparticles (0.2 wt%) and was denoted as WD_ZnO/ZnP_S, while another formulation contained aronia powder (0.1 wt%) in combination with ZnO_M.Coco.E nanoparticles (0.2 wt%), being denoted as WD_ZnO/ZnP_A. All concentrations were calculated relative to the total dry polymer mass.

Finally, a multi-bioactive hydrogel formulation incorporating all selected active agents was developed. In this case, spirulina powder (0.05 wt%) and aronia powder (0.05 wt%) were incorporated, resulting in a total botanical content of 0.1 wt%, together with ZnO_M.Coco.E nanoparticles (0.2 wt%), all values being reported relative to the total dry polymer mass. This formulation was denoted as WD_ZnO/ZnP_S_A.

For all formulations, the preparation procedure was identical. First, the required amount of botanical powder and/or ZnO/ZnP nanoparticles was added to the PVA solution. The powders were dispersed individually in 100 µL of deionized water, followed by ultrasonic bath treatment for 10 min to improve dispersion and homogeneity prior to incorporation into the PVA gel. Subsequently, the ALG solution was incorporated, and the resulting systems were mechanically stirred for 2 min to achieve a homogeneous distribution of all bioactive agents within the polymer matrix. The obtained hydrogels were ionically crosslinked using a 1% (*w*/*v*) CaCl_2_ aqueous solution. For physicochemical characterization, the crosslinked hydrogel dressings were frozen for 24 h and then lyophilized (freeze-dried) for 72 h. The detailed composition of the developed PVA/ALG hydrogel formulations is summarized in Table 6.

**Figure 24:**
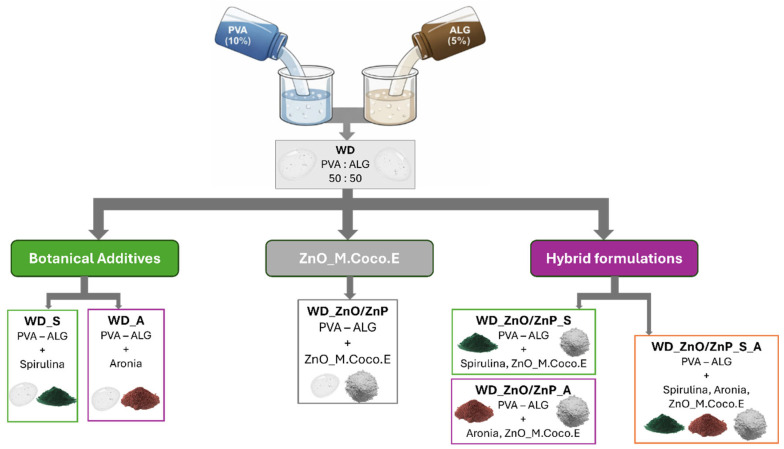
Schematic representation of each hydrogel wound dressing formulation.

**Figure 25:**
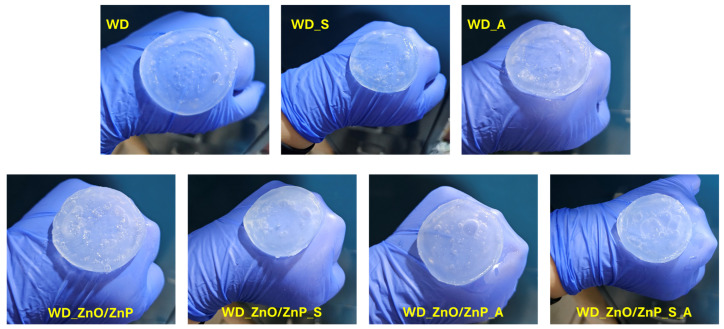
Macroscopic appearance of the developed hydrogel wound dressing formulations.

The composition of each hydrogel wound dressing formulation is schematically illustrated in Figure 24, while the macroscopic appearance of the developed hydrogels is presented in Figure 25.

All formulations were prepared using identical polymer concentrations and processing conditions; differences between samples arise exclusively from the type and amount of incorporated bioactive agents.

### 5.3. Characterization and Investigation Methods

The Zn-based powders were characterized by phase identification using a PANalytical Empyrean diffractometer (PANalytical, Almelo, The Netherlands). The setup included a hybrid monochromator (2×Ge 220) on the incident beam path and a parallel plate collimator on the diffracted beam path, combined with a PIXcel 3D detector. The structural characterization was conducted by grazing-incidence X-ray diffraction (GIXRD) at room temperature, with an incidence angle of ω = 0.5° and a 2θ scanning range of 5–80°. Cu Kα radiation (λ = 1.5406 Å) was applied with a current of 40 mA and a voltage of 45 kV. Powder morphology was analyzed by FIB–SEM with a Versa 3D microscope (Thermo Fisher Scientific, Waltham, MA, USA). The images were acquired using the secondary electron (SE) detector at an accelerating voltage of 30 keV. Elemental analysis was carried out using an energy-dispersive X-ray spectroscopy (EDS) system attached to the microscope. For the characterization of the wound dressing formulations, Infrared spectra were obtained by Fourier-transform infrared (FTIR) spectroscopy using a Thermo Scientific iN10-MX spectrometer (Thermo Fisher Scientific, Waltham, MA, USA) with a ZnSe crystal. Measurements were conducted at a resolution of 8 cm^−1^ with 32 scans over the spectral range 4000–400 cm^−1^. The wound dressings were also morphologically examined to assess structural modifications induced by the incorporation of various bioactive agents. Scanning electron microscopy (SEM) was performed using an Inspect F50 instrument (FEI, Hillsboro, OR, USA). Micrographs were collected using the backscattered electron (BSE) detector operated at 30 keV. To evaluate the swelling behavior of the formulations over time, the samples were cut into cylindrical specimens with a diameter of 5 mm. Each specimen was initially weighed to determine its dry mass and subsequently immersed in ultrapure water at room temperature. During the swelling experiment, samples were collected at defined time points (5, 30, 60, 180, 360, 720, and 1440 min). The surface liquid was removed with filter paper before weighing the specimens in order to determine their mass after immersion. The swelling ratio was subsequently calculated according to the following equation:

$$Swelling\;ratio=\frac{Wt-Wi}{Wi}\times 100\%$$

where *Wi* represents the dry mass of the formulation prior to liquid immersion, and *Wt* corresponds to the mass of the formulation after immersion at the specified time intervals.

Following the evaluation of the swelling rate, the degradation behavior of the dressings was assessed at the final immersion time point. After completion of the swelling experiment, the samples were withdrawn from the liquid medium and dried in an oven for 12 h. Subsequently, they were weighed in their dried state to determine the remaining mass. The degradation rate was determined using the following equation:

$$Degradation=(1-\frac{W0-Wt}{W0})\times 100\%$$

where *W*0 denotes the dry mass of the formulation prior to liquid immersion, and *Wt* represents the dry mass of the formulation measured after incubation in the liquid medium at the specified time points.

The influence of the developed wound dressing formulations on biofilm formation was evaluated against two pathogenic bacterial strains related to wound infections, namely the Gram-positive *Staphylococcus aureus* and the Gram-negative *Escherichia coli*. Prior to testing, the wound dressing samples (5 mm diameter disks) were sterilized by exposure to ultraviolet light for approximately 20 min. Sterile samples were individually placed in 24-well plates, and each well was filled with 1 mL of nutrient broth previously diluted 1:10 in sterile physiological saline. Bacterial suspensions adjusted to a 0.5 McFarland standard were used to inoculate the medium, resulting in a final concentration of about 10^6^ CFU/mL. The plates were incubated at 37 °C under static conditions for 24 and 48 h to allow bacterial adhesion and biofilm development on the surface of the dressings. After incubation, the culture medium was removed, and the samples were gently rinsed with sterile physiological saline solution in order to remove non-adherent (planktonic) bacterial cells. Subsequently, each specimen was placed into sterile 1.5 mL Eppendorf tubes containing 900 μL of sterile saline buffer. Subsequently, 100 μL of the corresponding well content was added, and the tubes were vortexed vigorously for approximately 20 s to detach the adhered bacterial cells from the dressing surface and obtain a homogeneous bacterial suspension. The suspensions were serially diluted tenfold in sterile saline. From each dilution, 10 μL aliquots were plated on nutrient agar and incubated at 37 °C for 24 h. Colony numbers were subsequently determined and reported as log_10_ CFU/mL. The antibacterial inhibition rate (%) was calculated relative to the bacterial control using the following equation:

$$Antibacterial\;rate\left(\%\right)=(1-\frac{CFUsample}{CFUcontrol})\times 100$$

In this calculation, CFUcontrol was considered as representing 100% bacterial viability (0% antimicrobial activity), while CFUsample corresponds to the colony-forming unit values obtained after testing against the two bacterial strains. In cases where CFUsample exceeded CFUcontrol, resulting in negative values indicative of bacterial growth promotion, these values were displayed as 0% inhibition in the graphical representation to ensure clarity.

The experiments were repeated twice, and the results are reported as the mean value ± standard deviation (SD). To evaluate the biological response to the developed wound dressings (WD), two cell lines were employed: human keratinocytes HaCaT and murine macrophages RAW 264.7. The human HaCaT cell line was used to assess the biocompatibility of the wound dressings, while macrophages were used to assess a potential pro-inflammatory response. Both cell lines were grown in Dulbecco’s Modified Eagle’s Medium (DMEM; Sigma-Aldrich, St. Louis, MO, USA) enriched with 10% fetal bovine serum (FBS, Gibco, Thermo Fisher Scientific, Waltham, MA, USA) and 1% penicillin-streptomycin mixture (Sigma Aldrich). Incubation was performed at 37 °C, in a humidified environment with 5% CO_2_.

Before biological testing, the wound dressing sample was UV-irradiated for 20 min to sterilize it, and the sample was aseptically transferred into sterile 48-well culture plates. For biocompatibility testing, HaCaT cells were seeded directly onto the surface of the materials at a density of 0.5 × 10^5^ cells/well. After allowing 30 min for initial cell attachment, the culture medium was carefully replenished to fully cover the samples.

Cell viability was evaluated by the MTT colorimetric assay, which relies on the conversion of 3-(4,5-dimethylthiazol-2-yl)-2,5-diphenyltetrazolium bromide (MTT; Sigma-Aldrich) into insoluble purple formazan crystals by metabolically active cells. After incubation with the tested materials for 24 and 72 h, the culture medium was removed and replaced with freshly prepared MTT solution (1 mg/mL in serum-free medium). The plates were then incubated for 4 h under standard cell culture conditions. Subsequently, the MTT solution was removed, and the resulting formazan crystals were dissolved in isopropanol. The absorbance was measured at 550 nm using a FlexStation 3 multimode microplate reader (Molecular Devices, San Jose, CA, USA). Cell viability was determined by normalizing the optical density (OD) values of each experimental group to the mean OD value of the control group at 24 h. To assess the cytotoxicity of the developed wound dressings, cell membrane integrity following 24 h and 72 h of cell-material contact was evaluated by quantifying lactate dehydrogenase (LDH) release into the culture medium. LDH is a stable cytosolic enzyme released into the extracellular environment upon loss of membrane integrity; therefore, its extracellular concentration is considered an indicator of cell membrane damage and cytotoxicity. Culture supernatants were harvested at the selected time points and analyzed using the LDH-based In Vitro Toxicology Assay Kit (TOX-7; Sigma-Aldrich, St. Louis, MO, USA) according to the manufacturer’s instructions. In brief, the collected medium was mixed with the reaction reagent and incubated for 30 min at room temperature in the dark. Absorbance was measured at 490 nm using a FlexStation 3 microplate reader.

To investigate the potential of the developed wound dressings to trigger an intrinsic pro-inflammatory response, the nitric oxide (NO) production was assessed in macrophage cultures. RAW 264.7 macrophages were seeded on the wound dressing materials at a density of 0.5 × 10^5^ cells/well. Following a 30 min attachment period, complete culture medium was carefully added to each well to cover the samples. Cells treated with lipopolysaccharide (LPS) from *Escherichia coli* O111/B4 (10 μg/mL; Sigma-Aldrich) served as the positive control for NO production. After 6 h and 24 h of contact with the tested materials, culture supernatants were collected for NO quantification, which was assessed indirectly by measuring nitrite accumulation using the Griess reagent system (Promega, Madison, WI, USA), according to the manufacturer’s protocol. Briefly, 50 μL of cell culture supernatant was mixed with an equal volume of sulfanilamide solution and incubated at room temperature in the dark. N-(1-naphthyl)ethylenediamine dihydrochloride (NED) solution was subsequently added. Upon completion of the reaction, the absorbance was recorded at 550 nm using a FlexStation III microplate reader (Molecular Devices, San Jose, CA, USA). Nitrite concentrations were determined based on a sodium nitrite calibration curve prepared according to the kit protocol. The results are expressed as mean ± standard deviation (SD) from three independent experiments.

Experiments were repeated three times, and the results are expressed as mean ± SD. Statistical analysis of the data was performed using GraphPad Prism (version 9), employing two-way ANOVA followed by Bonferroni’s multiple-comparison test. A value of *p* ≤ 0.05 was considered statistically significant.

## Figures and Tables

**Table 1:** EDS quantitative results obtained for ZnO_M.Coco, ZnO_M.Coco.E, ZnO_W.Coco after thermal treatment at 400 °C for 3 h.

	ZnO_M.Coco	ZnO_M.Coco.E	ZnO_W.Coco
Element	Weight %	Weight %	Weight %
C k	51.62	28.01	11.04
O k	29.87	41.02	28.78
P k	5.05	10.10	12.03
Zn k	13.46	20.88	48.15

**Table 2:** EDS quantitative results obtained for ZnO_M.Coco, ZnO_M.Coco.E, ZnO_W.Coco after thermal treatment at 800 °C for 2 h.

	ZnO_M.Coco	ZnO_M.Coco.E	ZnO_W.Coco
Element	Weight %	Weight %	Weight %
O k	41.28	54.40	53.25
P k	16.47	18.41	21.25
Zn k	42.25	27.19	25.50

**Table 3:** EDS quantitative results obtained for ZnO_M.Coco, ZnO_M.Coco.E, ZnO_W.Coco after thermal treatment at 550 °C for 2 h, followed by an additional treatment at 650 °C for 2 h.

	ZnO_M.Coco	ZnO_M.Coco.E	ZnO_W.Coco
Element	Weight %	Weight %	Weight %
C k	7.70	3.12	3.05
O k	51.70	59.51	50.60
P k	16.98	18.35	18.69
Zn k	23.62	19.02	27.66

**Table 4:** Wavenumbers at which the absorption bands were identified for each spectrum of all wound dressing formulations.

	Wavenumber (cm^−1^)												
WD	3323	2937	2916	1734	1606	1416	1373	1243	1086	1025	946	822	603
WD_S	3322	2937	2916	1734	1605	1416	1373	1243	1087	1025	947	821	603
WD_A	3312	2937	2916	1734	1605	1416	1373	1243	1086	1026	947	819	604
WD_ZnO/ZnP	3324	2938	2916	1733	1607	1417	1373	1242	1087	1024	946	822	603
WD_ZnO/ZnP_S	3314	2937	2915	1734	1605	1416	1373	1243	1088	1025	947	821	603
WD_ZnO/ZnP_A	3314	2937	2915	1734	1605	1415	1373	1243	1086	1025	946	819	604
WD_ZnO/ZnP_S_A	3314	2937	2916	1734	1606	1415	1373	1242	1086	1025	946	820	604

**Table 5:** Codes and synthesis origin of the ZnO nanoparticle samples obtained via green methods.

No.	Sample Code	Synthesis Source	Remarks
**1**	ZnO_M.Coco	Coconut milk	Raw commercial coconut milk used directly in the synthesis
**2**	ZnO_M.Coco.E	Coconut milk extract (centrifuged)	Fat removed by centrifugation prior to synthesis
**3**	ZnO_W.Coco	Coconut water	Clear coconut water filtered before use

**Table 6:** Composition of PVA/ALG-based hydrogel wound dressings.

Sample Code	PVA (10% *w*/*v*):ALG (5% *w*/*v*) (*v*/*v*)	Spirulina Powder (wt%) *	Aronia Powder (wt%) *	ZnO_M.Coco.E (wt%) *	Description
**WD**	50:50	-	-	-	Control hydrogel without bioactive agents
**WD_S**	50:50	0.10	-	-	WD containing spirulina powder
**WD_A**	50:50	-	0.10	-	WD containing aronia powder
**WD_ZnO/ZnP**	50:50	-	-	0.20	WD containing green synthesized ZnO/ZnP nanoparticles (ZnO_M.Coco.E)
**WD_ZnO/ZnP_S**	50:50	0.10	-	0.20	WD containing spirulina and ZnO/ZnP nanoparticles
**WD_ZnO/ZnP_A**	50:50	-	0.10	0.20	WD containing aronia and ZnO/ZnP nanoparticles
**WD_ZnO/ZnP_S_A**	50:50	0.05	0.05	0.20	Multi-bioactive WD containing spirulina, aronia, and ZnO/ZnP nanoparticles

* wt% values are reported relative to the total dry polymer mass (PVA + ALG).

## Data Availability

The data supporting the findings of this study are included within the article. Further information can be obtained from the corresponding author upon reasonable request.
